# Tailoring Biomass‐Derived Organosolv Lignin Derivatives for High‐Capacity Adsorption of Rhodamine B

**DOI:** 10.1002/cssc.202502472

**Published:** 2026-02-13

**Authors:** Sayantani Bhattacharya, Maxim Galkin, Michelle Åhlén, Maria Strømme, Johan Gising

**Affiliations:** ^1^ Division of Nanotechnology and Functional Materials Department of Materials Science and Engineering Uppsala University Uppsala Sweden

**Keywords:** adsorption, biomass, dyes/pigments, lignin, water purification

## Abstract

The valorization of biomass into renewable, high‐performance, adsorbent materials offers a sustainable alternative to conventional synthetic sorbents. In this study, we investigate the potential of lignin derivatives as efficient adsorbents for removing the cationic dye Rhodamine B (RhB) from aqueous solutions. Five organosolv lignin derivatives were synthesized via a one‐step process using phenol, catechol, resorcinol, pyrogallol, and hydroquinone as phenolic modifiers to introduce structural diversity. The influence of these modifications on the materials’ physicochemical properties and adsorption behavior was examined. Comprehensive characterization included ^31^P NMR, Brunauer–Emmet–Teller surface area analysis, size exclusion chromatography, thermogravimetric analysis, and dynamic light scattering. Among the derivatives, resorcinol‐modified lignin (**ReL**) showed the highest RhB adsorption capacity (101.2 mg g^−1^), attributed to its favorable textural properties—high surface area and pore volume—together with increased availability of functional groups, which collectively enhanced adsorption efficiency. Adsorption kinetics for all materials followed the pseudo‐second‐order model, indicating chemisorption as the dominant mechanism. Isotherm analyses revealed Langmuir‐type monolayer adsorption for **ReL**, pyrogallol‐modified, and hydroquinone‐modified lignins. Moreover, **ReL** demonstrated good recyclability, retaining 62% of its adsorption efficiency after five adsorption–desorption cycles. Collectively, these results highlight the promise of structurally engineered lignin‐based adsorbents as cost‐effective, efficient, and reusable materials for sustainable wastewater treatment.

## Graphical abstract

Organosolv lignin derivatives modified with phenolic compounds were synthesized via a one‐step lignin‐first approach and evaluated as renewable adsorbents for removing Rhodamine B dye from water. Among them, resorcinol‐modified lignin showed the highest adsorption capacity (101 mg g^−1^), driven by high surface area, pore volume, and accessible hydroxyl groups enabling efficient and reusable RhB capture.

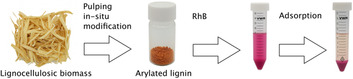

## 1 Introduction

Industrial wastewater contamination is a critical global issue that poses serious threats to both human health and aquatic ecosystems. Among the various pollutants, synthetic water‐soluble dyes are particularly alarming due to their carcinogenic potential, acute toxicity, and high environmental persistence [1]. Industries such as textiles, paints, paper, leather, and printing collectively discharge hundreds of dye types into the environment [2]. Each year, ≈7 × 10^7^ tons of synthetic dyes are produced worldwide, with the textile industry alone accounting for nearly 60% of the total [3]. It is further estimated that 10–15% of these dyes are discharged into aquatic systems through untreated or inadequately treated wastewater [2]. Even at low concentrations, dye‐polluted wastewater can severely impact aquatic ecosystems and human health. Most synthetic dyes are chemically stable, resistant to oxidation, and recalcitrant to biodegradation, leading to prolonged environmental persistence [4]. Their intense coloration reduces water transparency, limits sunlight penetration, and lowers dissolved oxygen levels, which significantly disrupts aquatic life [5]. Moreover, human exposure to dye‐contaminated water can cause dermatological problems, gastrointestinal disorders, and other health issues [6].

Among various synthetic dyes, Rhodamine B (RhB), a highly water‐soluble cationic xanthene dye, is extensively used in textile dyeing, paints, leather processing, ballpoint pen inks, dye lasers, stamp pads, and fireworks [7]. Due to its chemical stability and nonbiodegradability, RhB is considered one of the most hazardous textile dyes. It is carcinogenic and neurotoxic and can cause eye, skin, and respiratory irritation, as well as liver and thyroid damage after prolonged exposure [8]. In aquatic environments, RhB inhibits photosynthesis by blocking light penetration, thereby destabilizing aquatic ecosystems. Toxicological studies have shown its lethality to aquatic species such as sheepshead pupfish, with an LC_50_ value of 83.9 mg L^−1^ [9] Given its persistence, toxicity, and widespread industrial use, the sustainable and cost‐effective removal of RhB from aqueous media remains a pressing challenge in environmental remediation research. However, conventional treatment technologies such as coagulation, flocculation, chemical oxidation, and membrane filtration are often hindered by drawbacks including high operational costs, incomplete elimination of contaminants, and the generation of secondary waste streams [10, 11].

Among available techniques, adsorption stands out as a practical, efficient, and widely applicable approach due to its simple operation, low cost, and potential high removal efficiency [5]. Although extensive research has been conducted on adsorbents for wastewater purification, many materials exhibit high efficiency only under extreme pH or temperature conditions, which are rarely applicable in real‐world treatment systems [12, 13, 14]. An ideal adsorbent should combine affordability with high adsorption capacity, pose minimal ecological risks, and operate effectively under realistic conditions. Therefore, adsorbents derived from sustainable or bio‐based sources are particularly beneficial, as they help prevent further environmental harm.

In this context, lignin has recently gained considerable attention as a potentially sustainable adsorbent material [15, 16]. As a structural component of plant cell walls, lignin is generated in large quantities as a side stream of the pulp and paper industry and hydrolysis process [13]. Its abundance of functional groups, including phenolic hydroxyl, aliphatic hydroxyl, and carboxyl moieties, enables strong interactions with dye molecules through electrostatic attraction, ion exchange, and complexation [17, 18]. These properties make lignin an appealing and renewable candidate for dye removal applications. Nevertheless, technical lignins (such as kraft lignin) typically exhibit lower pollutant removal efficiency compared to commercial adsorbents [19].

To address these limitations, various lignin types including kraft lignin, alkali‐extracted lignin, and organosolv lignin have been explored, often in combination with chemical modifications such as esterification, sulfonation, amination, or incorporation into composite materials [14, 20, 21, 22, 23, 24, 25, 26]. Such modifications can significantly enhance both adsorption capacity and selectivity. For example, Du et al. developed quaternary ammonium grafted lignin capable of adsorbing 41.85 mg g^−1^ of RhB, whereas Li et al. synthesized magnetic lignin‐based hollow microspheres with an adsorption capacity of 17.62 mg g^−1^ [13, 27]. Among the different lignin variants, organosolv lignin offers several advantages for dye removal, since it is suitable for direct use without extensive purification or chemical modification [15]. This feature simplifies application and reduces processing costs. Previous systematic studies on the role of functional groups of lignin have demonstrated that the contents of aromatic and aliphatic hydroxyl groups critically influence adsorption performance [28]. It has also been shown that the textural properties of lignin‐based sorbents equally determine adsorption efficiency [2].

Therefore, the present work provides new insights by systematically correlating the surface characteristics of organosolv lignins, synthesized through simultaneous lignin fractionation and in situ functionalization, with their adsorption performance. In this study, five organosolv lignin derivatives were synthesized using phenol (**PhL**), catechol (**CtL**), resorcinol (**ReL**), pyrogallol (**PgL**), and hydroquinone (**HQL**), as modifying agents to introduce additional structural diversity. The sample names of the resulting derivatives are given in parentheses following each modifying agent. Comprehensive characterization was performed using standard spectroscopic and analytical techniques. The impact of these modifications on physicochemical properties and dye adsorption performance was systematically evaluated. Key attributes such as functional groups concentration, surface area, pore volume, thermal stability, and structural homogeneity were quantified and correlated with adsorption efficiency. This work provides valuable insights into the design of lignin‐based adsorbents and the efficient use of widely available biopolymer resources.

## 2 Results and Discussion

### 2.1 Characterization of the Organosolv Lignin Derivatives

**FIGURE 1:**
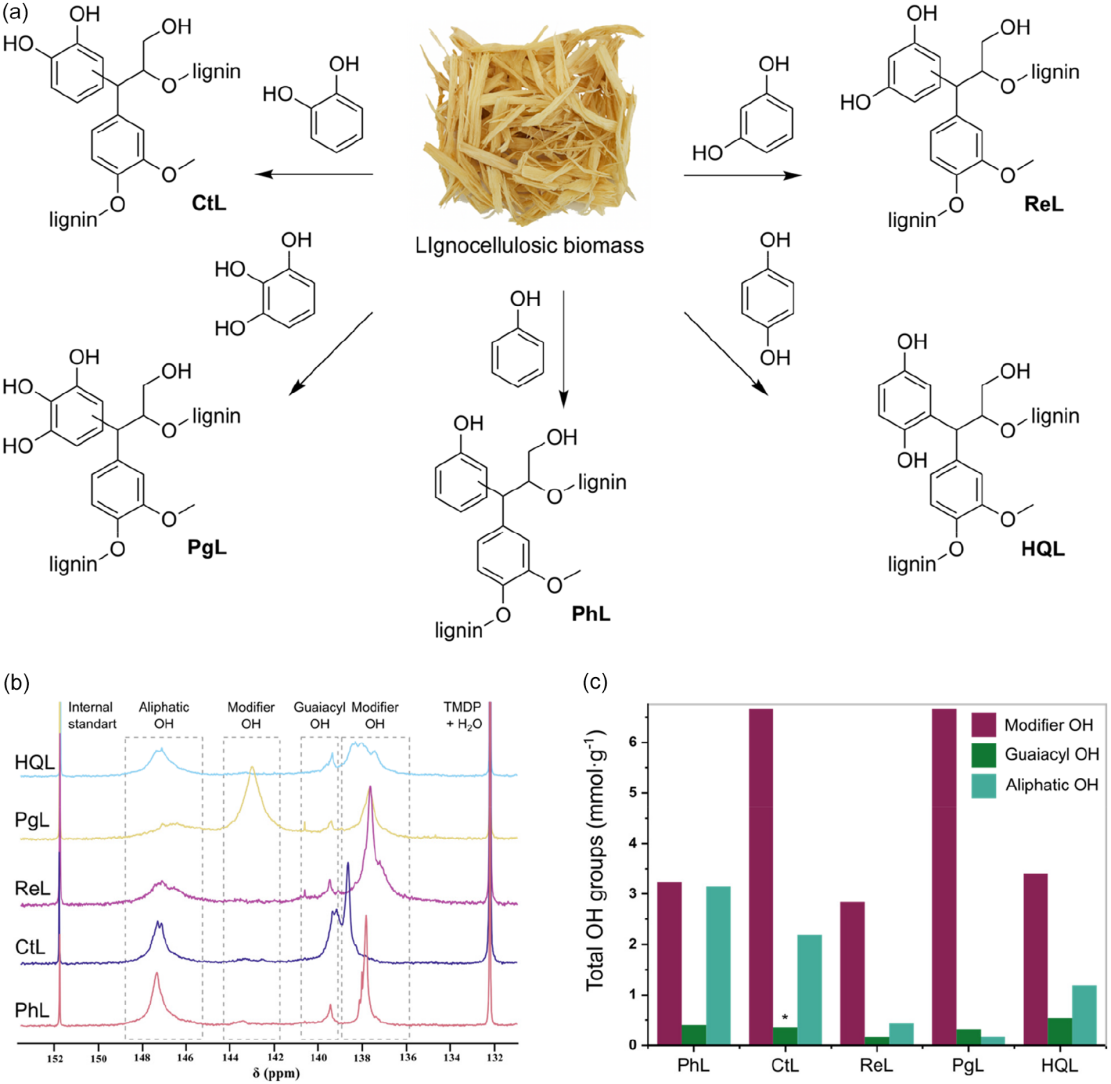
(a) Synthesis of organosolv lignin derivatives (**PhL**, **CtL**, **ReL**, **PgL**, and **HQL**) from lignocellulosic biomass; reaction condition: reflux in water for 5 h, using catalytic H_2_SO_4_ and the respective modifier, e.g., phenol, catechol, resorcinol, pyrogallol, and hydroquinone. (b) Comparative ^31^P NMR spectra of organosolv lignin materials—**PhL**, **CtL**, **ReL**, **PgL**, and **HQL** derivatized with TMDP using NHND as an internal standard. The signals within the chemical shift range marked by the box correspond to specific hydroxyl groups (OH) in various organosolv lignin derivatives; (c) total OH groups (mmol g^−1^) of different organosolv lignin derivatives. *The amount of guaiacyl unit was calculated from the average of other organosolv lignin derivatives. Synthesis and analysis were performed without replication.

With an objective to develop lignin‐based adsorbent materials from softwood biomass, we synthesized five distinct organosolv lignin derivatives using a lignin‐first biorefinery approach (Figure 1a). This one‐step process simultaneously integrates lignin extraction and functionalization during biomass fractionation (e.g., from sawdust) [29]. To examine how the arrangement of phenolic hydroxyl groups influences physicochemical properties and dye adsorption, phenol, catechol, resorcinol, pyrogallol, and hydroquinone were used as modifying agents, each possessing different numbers and positional arrangements of hydroxyl groups on the aromatic ring. The resulting organosolv lignin derivatives were characterized by ^31^P NMR spectroscopy, following the procedure by Meng et al. [30] to quantify the amount and type of hydroxyl groups (see Figure 1b,c and Supporting Information Table S3 and S4). Figure 1b displays distinct signals for aliphatic, guaiacyl, and modifier‐derived OH groups. As evident from Figure 1c, the choice of modifier during lignin extraction significantly influences the availability of different types of hydroxyl groups in the final material. The total hydroxyl content (aliphatic, guaiacyl, and modifier‐derived OH groups) varied among the lignin materials, with **CtL** having the highest amount of modifier OH groups (6.6 mmol g^−1^), **HQL** showing the highest guaiacyl OH content (0.54 mmol g^−1^), and **PhL** containing the greatest amount of aliphatic OH groups (3.15 mmol g^−1^).

Dynamic light scattering (DLS) was used to determine the hydrodynamic diameters of the lignin nanoparticles. The analysis revealed distinct particle size distributions across the samples (Figures S1–S5). **ReL** exhibited the smallest mean particle diameter (285 nm), whereas **PhL** showed the largest (1267 nm). The average particle size followed the order: **ReL** < **PgL** < **HQL** < **CtL** < **PhL**.

**FIGURE 2:**
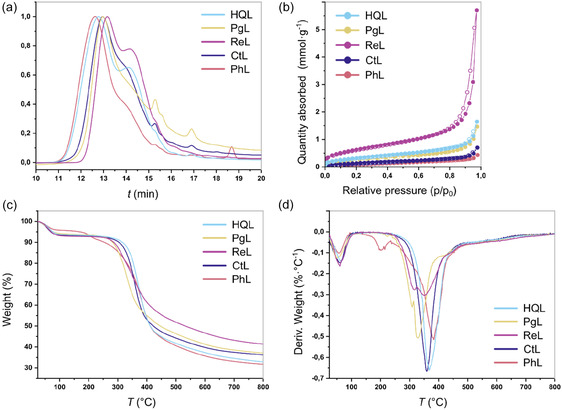
(a) Molecular weight distribution curves of organosolv lignin derivatives, **PhL**, **CtL**, **ReL**, **PgL**, and **HQL**, determined by SEC; (b) nitrogen adsorption/desorption isotherms for the organosolv lignin derivatives; and (c) TGA and (d) differential thermogravimetric analysis (DTG) curves of the organosolv lignin derivatives under nitrogen atmosphere.

Molecular weight is an important parameter of lignin, as it can influence the material's physicochemical properties [31, 32, 33]. The molecular weight distributions of the organosolv lignin derivatives were determined by size exclusion chromatography (SEC) analyses (Figure 2a). As summarized in Table S5, the number average molecular weights (*M*_*n*_) of the organosolv lignin derivatives ranged from 1800 to 2500 g·mol^−1^, while the weight‐average molecular weight (*M*_w_) ranged from 2400 to 4200 g mol^−1^. Among the prepared materials, **PhL** exhibited the highest molecular weights (*M*_w_ = 4200 g mol^−1^; *M*_*n*_ = 2500 g mol^−1^), whereas **ReL** displayed the lowest (*M*_w_ = 2400 g mol^−1^; *M*_*n*_ = 1800 g mol^−1^). The molecular weight trend of the organosolv lignin derivatives increased in the order **ReL** < **PgL** < **HQL** < **CtL** < **PhL**. Furthermore, the fractionation process provides lignin with low dispersity *Đ* (*M*_w_/*M*_*n*_ < 1.70), with **ReL** exhibiting the lowest dispersity (1.33) among the organosolv lignin derivatives obtained from softwood.

### 2.2 Analysis of Surface Area and Porosity

Adsorption capacity is strongly influenced by the physical attributes of sorbents, particularly their specific surface area, total pore volume, and pore size distribution, which are determined from nitrogen (N_2_) adsorption–desorption isotherms at 96°C [34, 35]. Figure 2b presents the N_2_ adsorption–desorption isotherms for the organosolv lignin derivatives, and Supporting Information Table S6 summarizes their Brunauer–Emmet–Teller (BET) specific surface areas (*SA*_BET_) and total pore volumes (*V*_tot_). The as‐synthesized organosolv lignin derivatives exhibit surface area values ranging from ≈9–52 m^2^ g^−1^, with **PhL** showing the lowest (9 m^2^ g^−1^) and **ReL** the highest (52 m^2^ g^−1^). Notably, these surface areas are rather high compared with other reported lignins in the literature, such as organosolv, alkali‐extracted, hydrolytically treated, and spruce lignins [14, 36, 37]. The DFT pore size distributions (Supporting Information Figure S6) revealed a combination of micropores (≤ 2 nm) and mesopores (2–50 nm), following IUPAC definitions [38]. Specifically, **ReL**, **PgL**, and **HQL** are dominated by micropores centered around 14–15 Å, while **PhL** and **CtL** contain mesopores near 27 Å.

The notably larger surface area of the organosolv lignin derivative, particularly **ReL**, can be attributed primarily to its enhanced microporosity [2, 39, 40]. The dominance of micropores substantially increases the BET surface area, as confirmed by recent studies demonstrating a strong correlation between micropore abundance and adsorption capacity [5, 40]. Furthermore, **ReL** also exhibits the largest total pore volume (0.20 cm^3^ g^−1^) among the synthesized organosolv lignin derivatives, which may enhance the accessibility of surface functional groups to adsorbate molecules, thereby promoting higher dye uptake. These trends are consistent with established understanding that high surface area and pore volume provide a greater number of adsorption sites and facilitate faster adsorption kinetics [41, 42, 43]. In contrast, **PhL** with its lower surface area (9 m^2^ g^−1^) and smaller pore volume (0.015 cm^3^ g^−1^) may restrict dye‐adsorbent interactions, leading to reduced adsorption efficiency.

### 2.3 Thermogravimetric Analysis

The thermal stability of adsorbents is a key determinant of their practical viability, especially in water treatment applications. Adsorbents used in water treatment must withstand operational or elevated temperatures without undergoing structural or functional degradation. In this context, lignin offers excellent thermal resilience owing to its highly aromatic backbone [44, 45]. The thermal decomposition profiles of the organosolv lignin derivatives, obtained between 25 and 800°C under N_2_ atmosphere, are depicted in the thermogravimetric analysis (TGA) and DTG curves (Figure 2c,d). The temperatures corresponding to 50% weight loss (*T*_d50%_), residual mass percentage at 800°C, and maximum mass‐loss rate temperature (*T*_dmax_) are presented in Supporting Information, Table S7. All samples exhibited an initial mass loss below 100°C, attributed to the desorption of physically adsorbed moisture. Due to the abundance of polar functional groups (such as hydroxyl groups), organosolv lignin derivatives readily absorb moisture from the surrounding atmosphere. Upon heating, this absorbed water desorbs at relatively low temperatures (<100°C), leading to the observed mass loss. No thermal degradation of the lignin backbone occurs in this temperature range. The dominant decomposition phase occurred between 200 and 500°C, with peak DTG temperatures of 383°C (**PhL**), 358°C (**CtL**), 350°C (**ReL**), 327°C (**PgL**), and 367°C (**HQL**), corresponding to the cleavage of aryl‐ether and C–C linkages, as well as the breakdown of phenolic units and aliphatic side chains [46]. Beyond 600°C, degradation slowed markedly, and the TGA curves approached a plateau above 700°C, indicating the conversion to residual char. At 800°C, char yields varied among the samples, with **ReL** retaining the highest residual mass (41%) and **PhL** the lowest (32%). The higher char formation tendency of **ReL** is likely due to the presence of a more condensed lignin structure, resulting in a broader and relatively slower mass loss rate as observed from the DTG curve [45]. Notably, **ReL** also displayed the highest T_d50%_ (around 530°C) among the samples, indicating enhanced thermal stability relative to the other lignin derivatives.

### 2.4 Dye Removal

#### 2.4.1 Adsorption Kinetics

**FIGURE 3:**
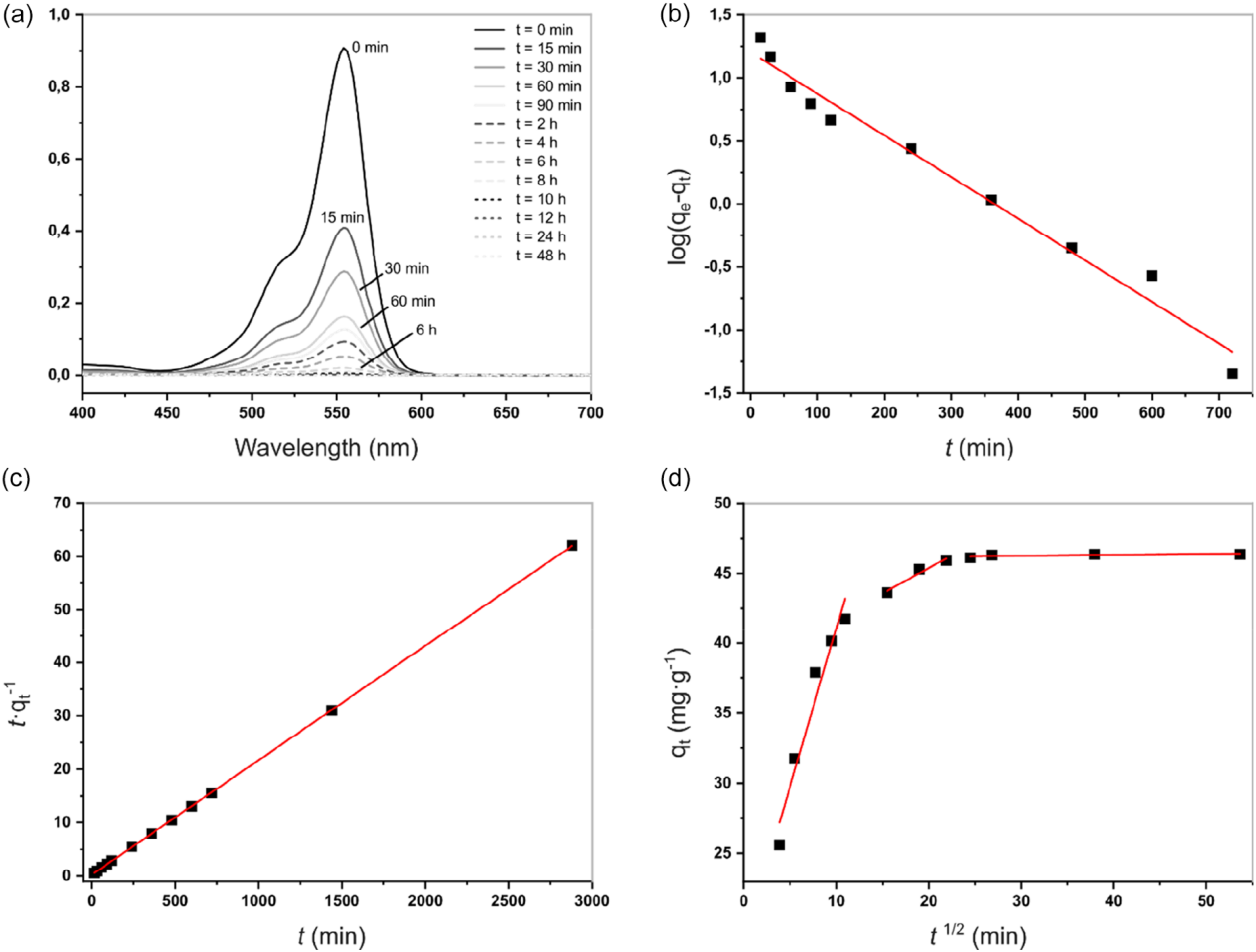
(a) Time‐dependent RhB absorption spectra using **ReL** as adsorbent, *C*_0_ = 500 ppm. Kinetic curves of (b) pseudo‐first‐order, (c) pseudo‐second‐order, and (d) intraparticle diffusion models.

Following the characterization of the key physical parameters that influence adsorption performance, we evaluated the efficiency of the organosolv lignin derivatives for dye removal from aqueous solution at 25°C. RhB was selected as a representative cationic dye, and its uptake by the organosolv lignin derivatives was investigated at neutral pH (7.0–7.2), representative of typical municipal and moderately treated textile wastewater. In the adsorption experiments, 500 mg of each organosolv lignin derivative was added to 50 mL of RhB solution (500 ppm), and dye uptake was monitored by recording UV‐Vis absorption spectra of the dye solution at predetermined time intervals. The effect of contact time on dye removal by the most effective material, **ReL**, is shown in Figure 3a, with corresponding results for **PgL**, **PhL**, **CtL**, and **HQL** provided in **Supporting Information, Figures S8a**–**11a**. It is evident that the amount of dye adsorbed in the first 15 min varied notably among the organosolv lignin derivatives. **ReL** achieved the highest early uptake, removing ≈ 55 % of the dye within the first 15 min, whereas **PgL** and **HQL** removed ≈45% and 34%, respectively. In contrast, **PhL** and **CtL** exhibited the lowest initial adsorption, with ≈11 and 12% removal, respectively. Although the adsorption rate for **ReL** and **PgL** gradually decreased after 6 and 12 h, respectively, both lignin derivatives achieved almost complete decolorization of the dye solutions within 24 h.

To elucidate the dye removal mechanism, experimental data collected at different time intervals were fitted to pseudo‐first‐order Equation (1), pseudo‐second‐order Equation (2), and Weber−Morris intraparticle diffusion Equation (3) models. The applicability of these models was validated by analyzing their corresponding correlation coefficients (*R* ^2^) [47, 48, 49].

(1) $$\log\;(q_{e}-q_{t})=\log\;q_{e}-\frac{k_{1}}{2.303}t$$

(2) $$\frac{t}{q_{t}}=\frac{1}{k_{2}{q_{e}}^{2}}+\frac{k_{1}}{2.303}t$$

(3) $$q_{t}=k_{i}\times t^{\frac{1}{2}}+C$$

where *q*_t_ (mg g^−1^) and *q*_e_ (mg g^−1^) are the amounts of the dye removed at time *t* and at equilibrium, respectively; *k*_1_ (min^−1^) is the pseudo‐first‐order rate constant, *k*_2_ (g mg^−1^ min^−1^) is the rate constant for the pseudo‐second‐order model, and *k*_i_ is the intraparticle diffusion rate constant.

The results obtained are depicted in Figure 3b–d, and Supporting Information Figures S8–S11b–d, Table 1. The high degree of linearity observed for the pseudo‐second‐order kinetic models across all the five organosolv lignin derivatives (*R* ^2^ = 0.994 for **PhL** and **CtL**; 0.999 for **ReL** and **PgL**; and 0.998 for **HQL**) indicates that the dye removal kinetics are best described by the pseudo‐second‐order model. These findings indicate that the dye removal process is predominantly governed by a chemisorption mechanism [2, 13, 27, 50, 51]. Moreover, the equilibrium adsorption capacities (*q*_e_) calculated from the pseudo‐second‐order model—11.5, 17.2, 46.6 ± 0.1, 46.5, and 34.6 mg g^−1^ for **PhL**, **CtL**, **ReL**, **PgL**, and **HQL,** respectively—closely matched the experimental values of 9.5, 16.1, 47.9, 46.4, and 31.3 mg g^−1^, thereby validating the model validity. Although the equilibrium capacities (*q*_e_) of **ReL** and **PgL** were similar under the examined kinetic conditions, **ReL** exhibited a substantially higher pseudo‐second‐order rate constant (*k*_2_ = (1.85 ± 0.24) × 10^−3^ g mg^−1^ min^−1^compared to **PgL** (k_2_ = 6.60 × 10^−4^ g mg^−1^ min^−1^). This result indicates a significantly faster adsorption rate for **ReL**, consistent with the experimental observations showing more rapid dye uptake for **ReL** than **PgL**.

**TABLE 1:** Kinetic parameters of RhB adsorption on PhL, CtL, ReL, PgL, and HQL.

Adsorbent	Pseudo‐first‐order model	Pseudo‐second‐order model	Intraparticle diffusion model
**PhL**	*R* ^2^ = 0.788	*R* ^2^ = 0.994	*R* ^2^ = 0.992
	*q*_e_ = 38.9 mg g^−1^	*q*_e_ = 11.5 mg g^−1^	*C* = 5.00 mg g^−1^
	*k*_1_ = (5.3 ± 0.86) × 10^−5^ min^−1^	*k*_2_ = (8.7 ± 1.9) × 10^−4^ g mg^−1^ min^−1^	*k*_i_ = 0.16 ± 0.01 mg g^−1^ min^−1^
**CtL**	*R* ^2^ = 0.728	*R* ^2^ = 0.994	*R* ^2^ = 0.992
	*q*_e_ = 37.2 mg g^−1^	*q*_e_ = 17.2 mg g^−1^	*C* = 3.69 mg g^−1^
	*k*_1_ = (1.1 ± 0.02) × 10^−4^ min^−1^	*k*_2_ = *(*4.4 ± 0.91) × 10^−4^ g mg^−1^ min^−1^	*k*_i_ = 0.49 ± 0.03 mg g^−1^ min^−1^
**ReL**	*R* ^2^ = 0.977	*R* ^2^ = 0.999	*R* ^2^ = 0.947
	*q*_e_ = 16.0 ± 2.36 mg g^−1^	*q*_e_ = 46.6 ± 0.07 mg g^−1^	*C* = 18.5 ± 2.43 mg g^−1^
	*k*_1_ = (7.6 ± 0.41) × 10^−3^ min^−1^	*k*_2_ = (1.9 ± 0.24) × 10^−3^ g mg^−1^ min^−1^	*k*_i_ = 2.3 ± 0.31 mg g^−1^ min^−1^
**PgL**	*R* ^2^ = 0.780	*R* ^2^ = 0.999	*R* ^2^ = 0.869
	*q*_e_ = 10.9 mg g^−1^	*q*_e_ = 46.5 mg g^−1^	*C* = 18.6 mg g^−1^
	*k*_1_ = (1.5 ± 0.25) × 10^−3^ min^−1^	*k*_2_ = (6.6 ± 0.08) × 10^−4^ g mg^−1^ min^−1^	*k*_i_ = 1.4 ± 0.28 mg g^−1^ min^−1^
**HQL**	*R* ^2^ = 0.7129	*R* ^2^ = 0.998	*R* ^2^ = 0.933
	*q*_e_ = 23.7 mg g^−1^	*q*_e_ = 34.6 mg g^−1^	*C* = 14.5 mg g^−1^
	*k*_1_ = (3.0 ± 0.59) × 10^−4^ min^−1^	*k*_2_ = (4.7 ± 0.85) × 10^−4^ g mg^−1^ min^−1^	*k*_i_ = 0.71 ± 0.08 mg g^−1^ min^−1^

The intraparticle diffusion plots for all organosolv lignin derivatives were multilinear, indicating the involvement of multiple adsorption stages rather than a single intraparticle diffusion–controlled process [49]. The first, steeper linear region corresponds to the diffusion of dye molecules from the bulk solution to the external surface of the lignin particles or boundary layer diffusion (Figure 3d). The second, less steep linear region corresponds to a gradual adsorption stage, during which intraparticle diffusion becomes the rate limiting step [4, 48]. The third region indicates the equilibrium phase, where adsorption sites become saturated and the system reaches stabilization. If the adsorption were governed solely by intraparticle diffusion, the plot of *q*_t_ versus *t* ^1/2^ would be rectilinear across the entire range and the lines would pass through the origin. However, in the case of the organosolv lignin derivatives, the plots did not pass through the origin (intercept, *c* ≠ 0), indicating that the intraparticle diffusion mechanism, although involved in the adsorption process, is not the sole rate‐limiting step governing the dye removal process [4].

#### 2.4.2 Adsorption Isotherm

To study the adsorption mechanism, experimental equilibrium adsorption capacities (*q*_e_) obtained at initial dye concentrations ranging from 50 to 1500 ppm were fitted to the Langmuir and Freundlich isotherm models. The Langmuir model, which assumes monolayer adsorption of the adsorbate molecules onto a homogeneous surface of the adsorbent with energetically equivalent binding sites, can be expressed as the following equation Equation (4) [47]

(4) $$\frac{c_{e}}{q_{e}}=\frac{1}{bq_{m}}+\frac{c_{e}}{q_{m}}$$

where *c*_e_ is the equilibrium concentration of the dye in mg L^−1^, *q*_e_ is the amount of the dye removed by the lignin materials in mg g^−1^, *q*_m_ is the monolayer adsorption capacity of the adsorbent in mg g^−1^, and *b* is the Langmuir constant.

The validity of Langmuir model can also be evaluated using the following dimensionless parameter (*R*_L_)

(5) $$R_{L}=\frac{1}{1+bc_{0}}$$

where *c*_0_ is the initial dye concentration in mg L^−1^, and the *R*_L_ value defines the adsorption type. Adsorption is irreversible if *R*_L_ = 0, linear for *R*_L_ = 1, unfavorable for *R*_L_ > 1, and favorable if 0 < *R*_L_ < 1.

The Freundlich model assumes that the adsorption occurs as a multilayer process onto a heterogeneous surface, and is expressed as Equation (6) [27]

(6) $$\ln\;q_{e}=\ln\;k_{f}+\frac{1}{n}\ln\;c_{e}$$

where *n* is the Freundlich constant and *k*_f_ is the constant associated with the adsorption capacity. These parameters can be calculated from the slope and intercept of the linear plot of ln *q*_e_ versus ln *c*_e_.

**FIGURE 4:**
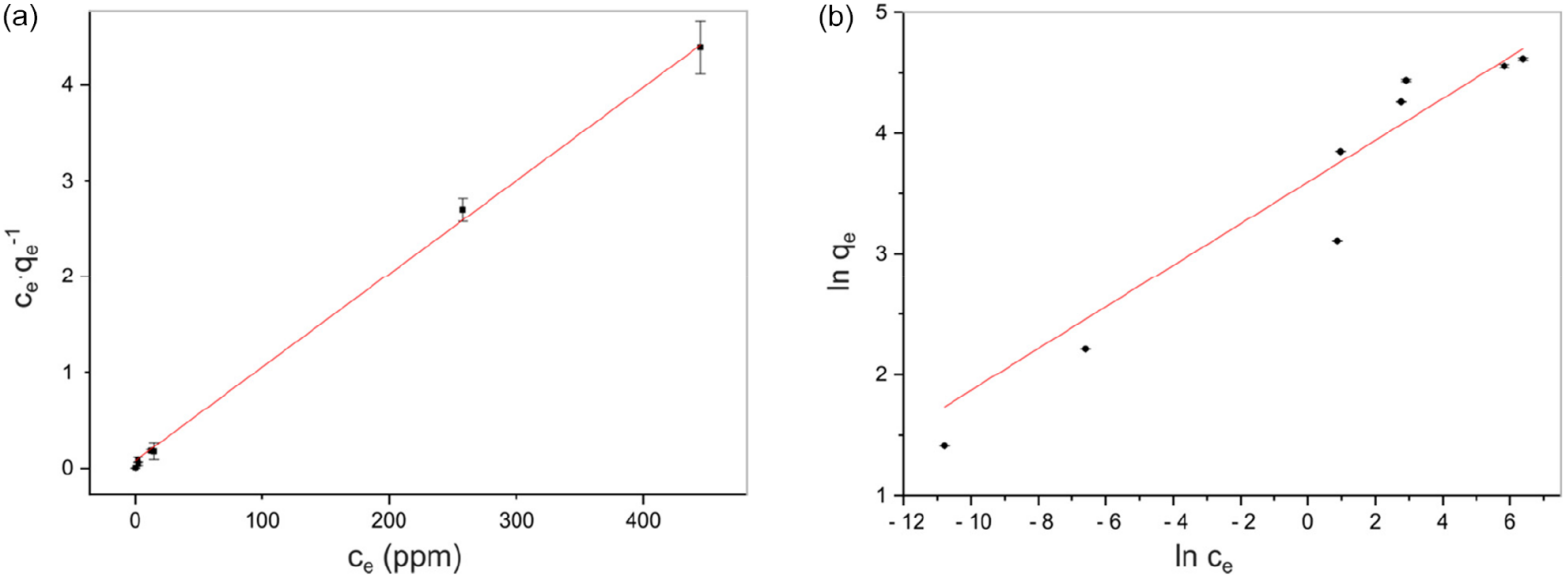
(a) Langmuir and (b) Freundlich adsorption isotherms for RhB adsorption onto ReL. All adsorption experiments were performed in triplicate (*n* = 3), and data points represent mean values. Parameter estimation was carried out by weighted least squares regression using propagated experimental uncertainties.

The adsorption isotherm fitting results are presented in Figure 4 and Supporting Information Figures S12–S15 and Table 2. For the three materials **ReL**, **PgL**, and **HQL**, the correlation coefficients for the Langmuir adsorption isotherms (R^2^ = 0.999, 0.996, and 0.987, respectively) were higher than that of the Freundlich isotherms (*R* ^2^ = 0.949, 0.869, and 0.931, respectively), indicating that the adsorption of RhB by these materials is more accurately described by the Langmuir model. Further, the *R*_L_ values for these materials fall between 0–1, confirming favorable Langmuir adsorption behavior. The superior applicability of the Langmuir isotherm suggests that RhB adsorption occurs predominantly through monolayer coverage on a relatively homogeneous lignin surface. Based on the Langmuir model, the maximum adsorption capacities (*q*_m_) were calculated to be 102.7 ± 1.3, 68.3, and 42.5 mg g^−1^ for **ReL**, **PgL**, and **HQL**, respectively.

**TABLE 2:** Langmuir and Freundlich isotherm parameters of RhB adsorption on PhL, CtL, ReL, PgL, and HQL. ReL related experiments were triplicated (parameter estimation for ReL was carried out by weighted least squares regression using propagated experimental uncertainties), all other experiments were performed without replication. Parameters uncertainties were taken from the standard errors of the regression.

Adsorbent	Langmuir model	Freundlich model
**PhL**	*R* ^2^ = 0.970	*R* ^2^ = 0.980
	*q*_m_ = 12.8 ± 0.90 mg g^−1^	*n* = 3.91 ± 0.23
	*R*_L_ = 0.07–0.70	
**CtL**	*R* ^2^ = 0.979	*R* ^2^ = 0.982
	*q*_m_ = 22.4 ± 1.3 mg g^−1^	*n* = 4.66 ± 0.26
	*R*_L_ = 0.05–0.63	
**ReL**	*R* ^2^ = 0.999	*R* ^2^ = 0.735
	*q*_m_ = 102.7 ± 1.3 mg g^−1^	*n* = 5.78 ± 1.41
	*R*_L_ = 0.005–0.134	
**PgL**	*R* ^2^ = 0.996	*R* ^2^ = 0.869
	*q*_m_ = 68.3 ± 1.70 mg g^−1^	*n* = 3.93 ± 0.62
	*R*_L_ = 0.006–0.171	
**HQL**	*R* ^2^ = 0.987	*R* ^2^ = 0.931
	*q*_m_ = 42.5± 1.90 mg g^−1^	*n* = 3.66 ± 0.41
	*R*_L_ = 0.025–0.437	

For the other two materials, **PhL** and **CtL**, the adsorption equilibrium data fitted well to both the Langmuir and Freundlich isotherm models. **PhL** showed R^2^ values of 0.970 (Langmuir) and 0.980 (Freundlich), whereas **CtL** exhibited 0.979 and 0.982, respectively. The comparable R^2^ values indicate that both models adequately describe the adsorption behavior. The slightly higher R^2^ for the Freundlich model (<1% difference) suggests minor surface heterogeneity, although the deviation is not statistically significant. Thus, dye adsorption on **PhL** and **CtL** likely proceeds through initial monolayer coverage on relatively uniform sites, followed by multilayer adsorption at higher concentrations, consistent with the slightly heterogeneous nature of lignin surfaces [2]. Similar dual‐model adsorption behavior has been reported for other adsorbents such as bagasse active carbon and biochar derived from pine cones, reflecting the coexistence of homogeneous and heterogeneous adsorption sites [52, 53].

**FIGURE 5:**
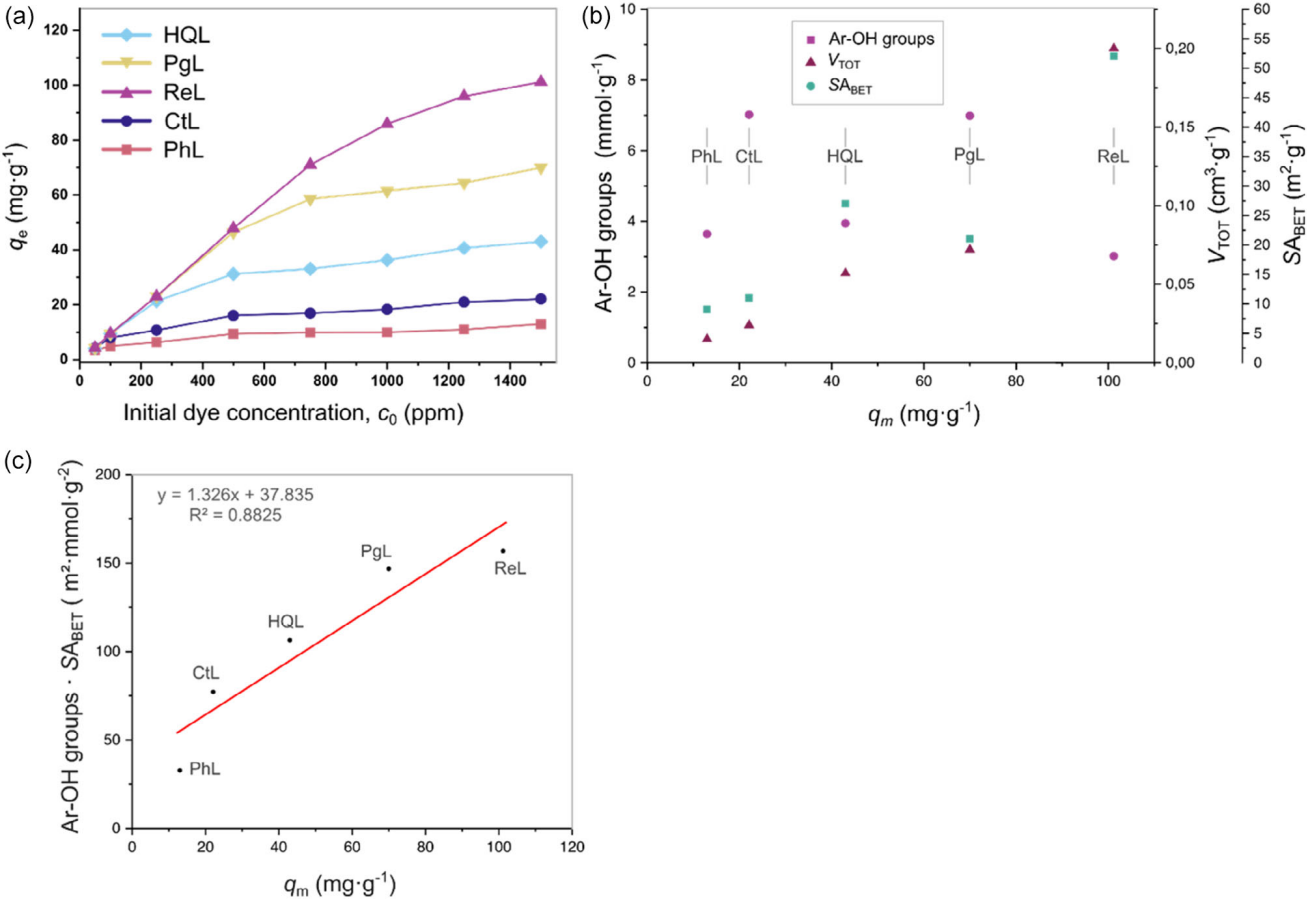
(a) Effect of initial concentration of RhB on adsorption onto **PhL**, **CtL**, **ReL**, **PgL**, and **HQL**. All experiments were performed without replication. (b) Modified lignins specific surface are (determined by BET), concentration of the total Ar‐OH groups (sum of modifier OH and guaiacyl OH groups measured through ^31^P NMR) or total pore volume versus maximum adsorption capacity; (c) modified lignins specific surface are (determined by BET) and concentration of the total Ar‐OH groups (sum of modifier OH and guaiacyl OH groups measured through ^31^P NMR) were compared to maximum adsorption capacity.

The equilibrium adsorption capacity of an adsorbent is strongly influenced by the initial dye concentration. As shown in Figure 5a, Rhodamine B uptake by all organosolv lignin derivatives increases sharply with increasing initial dye concentration. At lower concentrations, a large proportion of the lignin's active binding sites remained unoccupied, resulting in relatively low equilibrium adsorption capacities (*q*_e_). As the dye concentration increases, the greater concentration gradient enhances the driving force for mass transfer, enabling more dye molecules to access and occupy available adsorption sites, thereby leading to a rapid increase in *q*_e_. Beyond a certain concentration, however, the adsorption curves approached a plateau as the available binding sites became saturated, indicating that the adsorbent has reached its maximum adsorption capacity of these materials.

Interestingly, the equilibrium adsorption capacities (*q*_e_) varied substantially among the organosolv lignin derivatives. The maximum adsorption capacities followed the order: **PhL** (12.8 mg g^−1^) < **CtL** (22.4 mg g^−1^) < **HQL** (42.5 mg g^−1^) < **PgL** (68.3 mg g^−1^) < **ReL** (102.7 mg g^−1^). This trend can be rationalized by differences in the textural characteristics of the samples. **PhL**, with the lowest BET surface area (9 m^2^ g^−1^) and pore volume (0.015 cm^3^ g^−1^), possessed limited accessible surface area and internal porosity, restricting dye–adsorbent interactions and resulting in poor adsorption performance. In contrast, **ReL**, exhibiting the highest BET surface area (52 m^2^ g^−1^) and pore volume (0.20 cm^3^ g^−1^), offered substantially greater accessibility of functional groups and internal adsorption sites to dye molecules, thereby achieving the highest dye uptake. A clear linear increase in dye adsorption capacity with increasing pore volume was observed (Figure 5b), highlighting the role of pore accessibility in controlling adsorption behavior. Furthermore, **PgL**, despite having a lower surface area (21 m^2^ g^−1^) than **HQL** (27 m^2^ g^−1^), exhibited a higher pore volume (0.072 cm^3^ g^−1^ vs. 0.057 cm^3^ g^−1^), which enhanced dye diffusion and resulted in greater adsorption capacity than **HQL**. These findings confirm that both surface area and pore volume play critical roles in governing dye adsorption, consistent with the widely reported relationship that increased pore volume enhances accessible internal surface area, facilitating dye penetration, adhesion, and binding [54].

While textural properties strongly influence the accessibility of adsorption sites, the intrinsic chemical functionality of lignin—particularly the abundance and reactivity of surface hydroxyl groups—plays an equally important role in determining adsorption capacity**.** Adsorption capacity depends on both the number of active sites (e.g., acidic hydroxyl groups such as aromatic OH) and their accessibility for binding, which is largely governed by surface area (Figure 5b,c), consistent with previous observations. The incorporation of different chemical modifiers during lignin extraction introduced distinct structural variations within the lignin framework, thereby altering the distribution and availability of active functional groups. As a result, samples possessing larger surface area and higher concentrations of accessible phenolic hydroxyl groups exhibited the greatest equilibrium adsorption capacities, confirming that both surface morphology and chemical functionality act synergistically to enhance dye uptake.

### 2.5 FT‐IR Analysis

**FIGURE 6:**
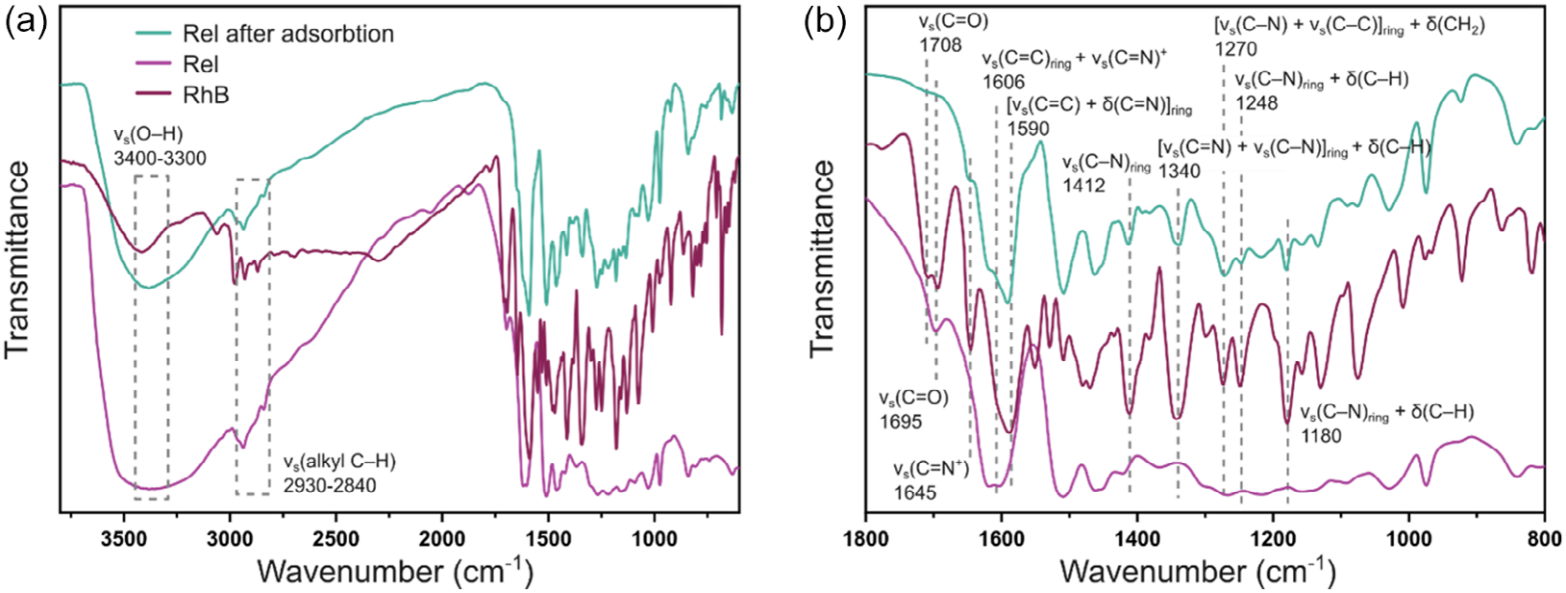
Comparative FT‐IR spectra of RhB and **ReL**, before and after adsorption (a) entire spectral range; and (b) enlarged view of key absorption bands.

Fourier transformed infrared spectroscopy (FT‐IR) spectra of RhB and **ReL** before and after adsorption are presented in Figure 6 and reveal notable spectral changes. The broad O–H stretching band of **ReL** within the 3300–3400 cm^−1^ range decreased in intensity postadsorption, indicating strong hydrogen bonding interactions between lignin hydroxyl groups and the amine functionalities of the dye. The aliphatic C–H stretching bands at 2930–2840 cm^−1^ exhibited slight intensity changes, suggesting partial overlap with the alkyl chains of RhB. Moreover, the aromatic C = C stretching band at around 1606 cm^−1^ shifted and overlapped with the RhB C = C stretching peak at 1590 cm^−1^, indicating the *π*–*π* stacking interactions between the lignin aromatic rings and the xanthene core of RhB. Further, concomitant attenuation of the conjugated C = O vibration of **ReL** at 1695 cm^−1^ and the C = N^+^(CH_3_)_2_ bands of RhB at 1645 cm^−1^ underscores substantial interactionbetween the dye molecules and the lignin framework [55]. In the fingerprint region, new peaks emerged at 1412, 1340, 1270, 1180, and 1248 cm^−1^, corresponding to aromatic C–N, C = N, aromatic ring, and amine‐bearing aromatic ring vibrations of RhB, strongly supporting successful dye incorporation onto the lignin surface [56]. These spectral modifications collectively validate that dye adsorption onto **ReL** proceeds predominantly through hydrogen bonding and *π*–*π* stacking interactions.

### 2.6 Desorption and Reusability

**FIGURE 7:**
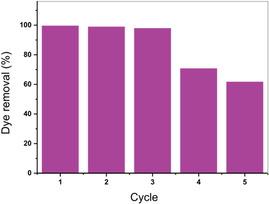
Recyclability test of **ReL**, adsorption of the dye RhB up to five cycles.

Incineration is a viable end‐of‐life scenario for bio‐based water‐treatment adsorbents. However, regeneration can be more beneficial, as it improves reusability and minimizes environmental impact [57]. Therefore, desorption of RhB was investigated using various eluting solvents for **ReL**, as it exhibited the highest dye uptake. Recyclability tests of lignin‐based materials often face substantial obstacles due to the intrinsic solubility of lignin in various solvents [58]. In general, organic solvents such as methanol and ethanol are frequently used for effective desorption of RhB, owing to the dye's high solubility in these solvents [59]. However, their use led to partial or complete dissolution of the lignin materials. Similarly, treatment with NaOH solutions (0.1–1.0 M) also caused partial to complete dissolution of the lignin material, while HCl solutions (0.1–0.5 M) led to incomplete dye removal from the adsorbent. The use of other organic solvents such as chloroform and dichloromethane was also ineffective for dye desorption. Given these limitations, ultrasonication at 50°C in distilled water was selected as an effective, environmentally friendly regeneration method. The washing step was continued until the supernatant solution became colorless, ensuring maximal dye desorption. Thereafter, the washed adsorbent was dried under vacuum at 70°C overnight prior to reuse in subsequent adsorption cycles (Figure 7). The dye removal efficiency remained nearly constant to the third cycle (from 99.7% to 98%). However, after the third cycle the dye removal efficiency declined to 71% in the fourth cycle and ~ 62% in the fifth cycle. This decrease in dye uptake during later cycles is attributed to a reduction in active adsorption sites, incomplete dye desorption, and physical degradation of the adsorbent during repeated regeneration. Compared with literature‐reported biobased adsorbents, the materials exhibit competitive recyclability. Several systems show substantial performance loss within 3–5 cycles, including Fe–N co‐modified biochar (~40% after five cycles^)^, sulfur‐doped biochar (~70% after five cycles), chitosan‐based composites (~60% and ~ 35% after three cycles), plant biomass adsorbents (~20% reduction over five cycles), and furfural‐residue‐based adsorbents, which showed a decrease from 95.0% to 85.6% by the fifth cycle [60, 61, 62, 63, 64]. In contrast, the organosolv lignin derivative retains 98% efficiency after three cycles and remains moderately effective by the fifth cycle, demonstrating strong overall reusability.

**FIGURE 8:**
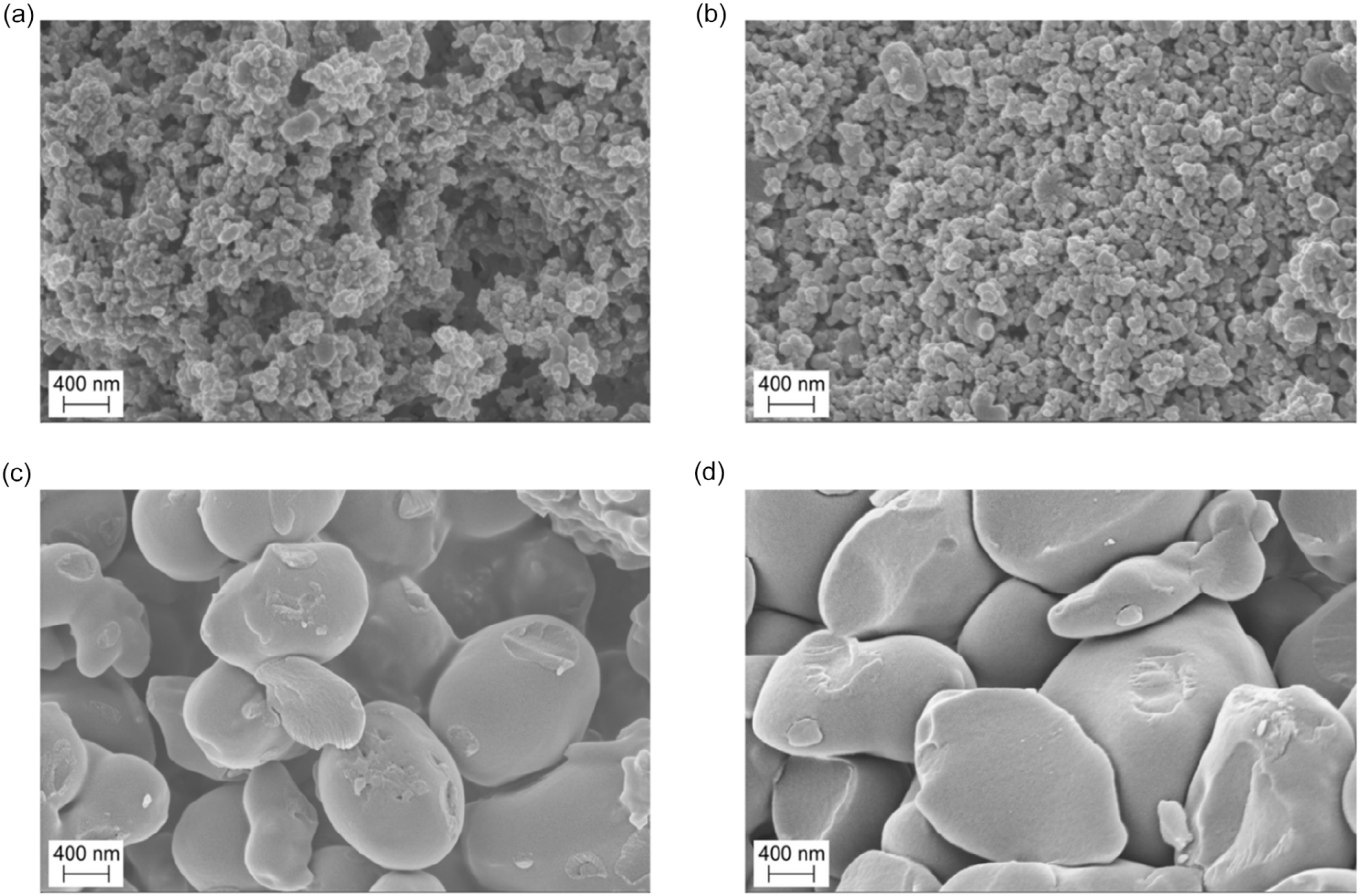
Field emission scanning electron microscopy images of **ReL** (a) before adsorption; (b) after the first cycle of adsorption of RhB; (c) after the third cycle of adsorption of RhB; and (d) after the fifth cycle of adsorption of RhB.

Morphological analysis via SEM (Figure 8) further supports these findings. After the first adsorption–desorption cycle, the nanoparticles retained their characteristic rough, textured surface morphology, with a mean particle diameter centered around 80 nm. This preservation of morphology correlates well with the recyclability data, which showed nearly unchanged dye adsorption capacity in the second cycle. However, substantial morphological changes were observed after the third and fifth cycles. The nanoparticles underwent pronounced coalescence, forming larger and smoother aggregates with a significantly increased average size (~1200 nm). The progressive morphological degradation aligns with the observed decline in dye adsorption efficiency in the later cycles, suggesting that nanoparticle aggregation and surface smoothing reduce the number of accessible active sites and hinder dye–surface interactions.

The adsorption capacity of adsorbents for dye removal varies widely and can reach, for example, values as high as *q*_m_ = 1859 mg g^−1^ for methylene blue using a nanocomposite of chitosan‐grafted poly(acrylic acid) and montmorillonite [65]. A comparison with biomass‐derived adsorbents reported in the literature (Table 3) reveals that the organosolv lignin derivative (**ReL**) prepared in this work exhibits a relatively high adsorption capacity for RhB along with distinct practical advantages. Some adsorbents, such as acid‐treated banana peel and furfural residue, achieve high dye removal only under extremely acidic conditions, which are unrealistic for most real‐world water treatment applications [64, 66]. On the other hand, other adsorbents require harsh preparation procedures, including high‐temperature carbonization [50, 67, 68, 70, 72]. In contrast, **ReL** demonstrated substantially higher adsorption capacity than untreated or slightly chemically modified biomass derived adsorbents, thereby demonstrating its effectiveness and suitability for dye removal from aqueous solutions under mild and environmentally relevant conditions.

**TABLE 3:** Adsorption capacities of various biomass‐derived adsorbents with their preparation methods for RhB removal.

Adsorbent	Preparation	*q*_m_ (mg g^−1^)	Ref
Banana peel	H_3_PO_4_ treatment	9.5	Oyekanmi et al. [66]
C. edulis plant	HCl treatment	90.90	Dabagh et al. [63]
Olive biomass waste	Activation with ZnCl_2_ and carbonized at 600°C	263.71	Albanio et al. [67]
Activated carbon from lignocellulosic waste	Impregnation with H_3_PO_4_ and CaCl_2_ followed by carbonization at 500°C	33.3	Silva Lacerda et al. [68]
Furfural residue	Untreated	37.93	Chen et al. [64]
Citric acid‐modified furfural residue biochar	Hydrothermal carbonization followed by citric acid modification	39.46	Li et al. [50]
Lignin‐Based Hollow Microspheres	Esterification of organosolv lignin with maleic anhydride	17.62	Li et al. [27]
Sawdust	Untreated	7.309	Suteu et al. [69]
Crosslinked lignin aerogels	Freeze‐drying of lignin followed by annealing at 300°C	156.4	Chen et al. [70]
Lignin‐modulated magnetic negatively charged Fe_3_O_4_@lignin/phenolic resins nanospheres	Reverse‐phase emulsion polymerization	41.03	Chen et al. [71]
Quaternized lignin (QL)	Quaternization of lignin	41.85	Du et al. [13]
Lignin‐derived carbon nanosheet (L‐CNS)	Freeze‐drying followed by high‐temperature carbonization.	41.2	Chen et al. [72]
Organosolv lignin (**ReL**)	Chemical Modification during extraction from lignocellulose	101.2	This work

## 3 Conclusions

In this study, organosolv lignin derivatives were synthesized via a streamlined, one‐step lignin‐first method, enabling simultaneous biomass fractionation and in situ lignin functionalization. Five distinct organosolv lignin derivatives were prepared using phenol, catechol, resorcinol, pyrogallol, and hydroquinone as phenolic modifiers to introduce structural diversity through varied hydroxyl group arrangements. The influence of these structural modifications on the physicochemical properties and dye adsorption performance was systematically investigated. Among the synthesized materials, **ReL** exhibited the highest RhB adsorption capacity (101.2 mg g^−1^), attributed to its favorable textural properties, i.e. high surface area and pore volume, in combination with chemical functionality, which enhances the accessibility and interaction of adsorption sites. The adsorption kinetics for all materials followed the pseudo‐second‐order model, indicating chemisorption as the dominant mechanism. The equilibrium adsorption data for **ReL**, **PgL**, and **HQL** were best described by the Langmuir isotherm, suggesting monolayer adsorption on homogeneous surfaces. Moreover, **ReL** demonstrated good recyclability, maintaining high adsorption efficiency over three consecutive cycles and remaining functional through five reuse cycles. While this study focuses on single‐solute systems, the selectivity of organosolv lignin derivatives toward RhB under competitive conditions remains an important topic for future investigation. Overall, this work underscores the potential of structurally engineered organosolv lignin‐based materials as sustainable, high‐performance adsorbents for wastewater treatment applications.

## 4 Experimental Section

Spruce sawdust (*Picea abies*, 40–80 years old) harvested in Svealand, Sweden (2023) was used as the biomass source. The sawdust was air‐dried prior to use. All chemicals, including phenolic modifiers and RhB, were analytical grade and used as received.

### 4.1 General Protocol for Synthesis of the Organosolv Lignin Derivatives from Biomass

Synthesis of the organosolv lignin derivatives was carried out following previously described procedure with minor modifications [29]. Briefly, in a three‐neck round‐bottom flask (3 L) equipped with mechanical PTFE propeller stirrer, required amount of dried biomass (300–400 g) and the phenolic modifier was presoaked overnight in water. The ratio of biomass to solvent was 1:5 w/w (stated solvent ratio includes both modifier and water). And, for example, resorcinol to water ratio was 1:1 w/w. After presoaking, catalytic amount of 12 M H_2_SO_4_ (2 wt% of the total solvent amount) was added and the mixture was heated for about 1 h until the temperature reached 100°C. Then, the reaction mixture was refluxed for 5 h. The reaction mixture was cooled down to 50–60°C and quenched by addition of methanol. The pulp was filtered off and washed with acetone using vacuum filtration. Lignin was isolated using an anti‐solvent method. For detailed procedure, see Supporting Information.

### 4.2 General Protocol for Adsorption Studies

Adsorption studies were performed using RhB as a model cationic dye at neutral pH (7.0–7.2). Kinetic experiments were conducted by mixing 500 mg of organosolv lignin derivative with 50 mL of RhB solution (500 ppm) and agitating the mixture in an orbital shaker (INC/REFRIG 5000IR) at 25°C and 130 rpm; 2 mL aliquots were collected at predetermined intervals and phase separation was achieved by centrifugation at 3800 × g for 10 min. Adsorption isotherm studies were carried out using 100 mg of adsorbent and RhB solutions with initial concentrations ranging from 50 to 1500 ppm, shaken under identical conditions for 18 h to reach equilibrium. Residual RhB concentrations were determined using an Agilent Cary 60 UV–vis spectrophotometer at 554 nm (400–700 nm scan range, 600 nm min^−1^ scan rate). The kinetic data were fitted (Origin 2019) to pseudo‐first‐order, pseudo‐second‐order, and Weber–Morris intraparticle diffusion models, while equilibrium data were analyzed using Langmuir and Freundlich isotherm models. Uncertainty parameters were estimated from the standard error of the linear regression.

## Supporting Information

Additional supporting information can be found online in the Supporting Information section. **Supporting Fig. S1:** Intensity‐weighted particle size distribution of PhL measured by DLS at room temperature. **Supporting Fig. S2:** Intensity‐weighted particle size distribution of CtL measured by DLS at room temperature. **Supporting Fig. S3:** Intensity‐weighted particle size distribution of **ReL** measured by DLS at room temperature. **Supporting Fig. S4:** Intensity‐weighted particle size distribution of **PgL** measured by DLS at room temperature. **Supporting Fig. S5:** Intensity‐weighted particle size distribution of HQL measured by DLS at room temperature. **Supporting Fig. S6:** Pore‐size distributions of **PhL**, **CtL**, **ReL**, **PgL**, and **HQL** obtained from BET analysis. **Supporting Fig. S7:** Calibration curve of the Rhodamine B dye. **Supporting Fig. S8:** (a) Time‐dependent Rhodamine B absorption spectra using **PgL** as adsorbent, *C*_0_ = 500 ppm. Kinetic curves of (b) pseudo‐first‐order, (c) pseudo‐second‐order, and (d) intraparticle diffusion models. **Supporting Fig. S9:** (a) Time‐dependent Rhodamine B absorption spectra of PhL, *C*_0_ = 500 ppm. Kinetic curves of (b) pseudo‐first‐order, (c) pseudo‐second‐order, and (d) intraparticle diffusion model. **Supporting Fig. S10:** (a) Time‐dependent Rhodamine B absorption spectra of **CtL**, *C*_0_ = 500 ppm. Kinetic curves of (b) pseudo‐first‐order, (c) pseudo‐second‐order, and (d) intraparticle diffusion models. **Supporting Fig. S11:** (a) Time‐dependent Rhodamine B absorption spectra of **HQL**, *C*_0_ = 500 ppm. Kinetic curves of (b) pseudo‐first‐order, (c) pseudo‐second‐order, and (d) intraparticle diffusion models. **Supporting Fig. S12:** (a) Langmuir and (b) Freundlich adsorption isotherms of Rhodamine B adsorption onto **PhL**. **Supporting Fig. S13:** (a) Langmuir and (b) Freundlich adsorption isotherms of Rhodamine B adsorption onto **CtL**. **Supporting Fig. S14:** (a) Langmuir and (b) Freundlich adsorption isotherms of Rhodamine B adsorption onto **HQL**. **Supporting Fig. S15:** (a) Langmuir and (b) Freundlich adsorption isotherms of Rhodamine B adsorption onto **PgL**. **Supporting Fig. S16:** Photos used for TOC; from left to right: spruce sawdust, isolated lignin, lignin plus 500 ppm RhB water solution before and after adsorption. **Supporting Fig. S17:** Sorption capacity expressed as specific surface are (determined by BET) and concentration of the total Ar‐OH groups (sum of modifier OH and guaiacyl OH groups measured through 31P NMR) vs maximum adsorption capacity. **Supporting Table S1**: Summary of the reaction conditions for synthesis of the organosolv lignin derivatives from biomass. **Supporting Table S2:** Wood composition analysis. **Supporting Table S3:** Different regions for the hydroxyl groups obtained from ^31^P NMR spectra of the organosolv lignin derivatives. **Supporting Table S4:** Quantitative amounts of hydroxyl groups in organosolv lignin derivatives determined from different regions of the ^31^P NMR spectra. **Supporting Table S5:** Number average molecular weight (*M*_n_), weight average molecular weight (*M*_w_), and dispersitie**s**, *Đ* (*M*_n_/*M*_w_) of organosolv lignin derivatives. **Supporting Table S6:** BET surface areas and total pore volumes obtained from N_2_ isotherms recorded at ‐96°C for the organosolv lignin derivatives. **Supporting Table S7:** Values of T_d50%_ (°C), Residue (%) and T_max_ (°C) for the lignin derivatives.

## Funding

This study was supported by Swedish Research Council (2020−00207), Carl Tryggers stiftelse för vetenskaplig forskning (23−02438), Åforsk (23−337) and Formas (2022−02042).

## Conflicts of Interest

The authors declare following competing financial interest(s): MG and JG are inventors on a Patent Application (WO2024231457A1, November 14, 2024) that covers the preparation and isolation of functionalized lignins via phenol‐assisted process. The patent application is owned by AB Karl Hedin Bio Innovation. MS, MG, and JG are partially involved in AB Karl Hedin Bio Innovation activities.

## Supporting information

Supplementary Material

## Data Availability

The data that support the findings of this study are available from the corresponding author upon reasonable request.
