# Supplementary material for: Tailoring Biomass‐Derived Organosolv Lignin Derivatives for High‐Capacity Adsorption of Rhodamine B
Source: ChemSusChem. 2026 Feb 13;19(4):e202502472. doi: 10.1002/cssc.202502472 (PMC12904731; doi:10.1002/cssc.202502472)
Supplement: Supplementary file 1 — Supplementary Material [file CSSC-19-e202502472-s001.pdf]

Tailoring Biomass-Derived Organosolv Lignin Derivatives for High-Capacity Adsorption of Rhodamine B

Sayantani Bhattacharya, Maxim Galkin*, Michelle Åhlén, Maria Strømme and Johan Gising*

Dr S. Bhattacharya
Dr M. Galkin
Dr M. Åhlén
Prof Dr M. Strømme
Dr J. Gising
Division of Nanotechnology and Functional Materials
Department of Materials Science and Engineering
Uppsala University
Ångströmlaboratoriet, Lägerhyddsvägen 1, 751 03 Uppsala, Sweden
E-mail: maxim.galkin@angstrom.uu.se, johan.gising@angstrom.uu.se

Table of Contents

Materials 4

General protocol for synthesis of the organosolv lignin derivatives from biomass (PhL, CtL, ReL, PgL, and HQL) 4

Table S1: Reaction conditions for the preparation of the organosolv lignin derivatives _ ***Error! Bookmark not defined.***

Table S2: Wood composition analysis 6

Determination of hydroxyl groups in lignin by quantitative ^{31}P NMR 6

Table S3: Different regions for the hydroxyl groups obtained from ^{31}P NMR spectra of the organosolv lignin derivatives 7

Table S4: Quantitative amounts of hydroxyl groups in organosolv lignin derivatives determined from different regions of the ^{31}P NMR spectra 8

Thermogravimetric Analysis (TGA) 8

Size Exclusion Chromatography 8

Table S5. Number average molecular weight (M_n), weight average molecular weight (M_w), and dispersities, Đ (M_n/M_w) of organosolv lignin derivatives. 9

Dynamic Light Scattering 9

FT-IR 9

Gas sorption 9

Table S6. BET surface areas and total pore volumes obtained from N_2 isotherms recorded at -96 °C for the organosolv lignin derivatives. 10

Kinetics and adsorption studies 10

Regeneration of the adsorbent 11

WILEY-VCH

SEM ______ *11*

Table S7: Values of $T_{d50\%}$ (°C), Residue (%) and T_{max} (°C) for the lignin derivatives ______ *11*

Figure S1: Intensity-weighted particle size distribution of PhL measured by DLS at room temperature. ______ *12*

Figure S2: Intensity-weighted particle size distribution of CtL measured by DLS at room temperature. ______ *12*

Figure S3: Intensity-weighted particle size distribution of ReL measured by DLS at room temperature. ______ *13*

Figure S4: Intensity-weighted particle size distribution of PgL measured by DLS at room temperature. ______ *13*

Figure S5: Intensity-weighted particle size distribution of HQL measured by DLS at room temperature. ______ *14*

Figure S6: Pore-size distributions of PhL, CtL, ReL, PgL, and HQL obtained from BET analysis. ______ *14*

Figure S7: Calibration curve of the Rhodamine B dye. ______ *15*

Figure S8. (a) Time-dependent Rhodamine B absorption spectra using PgL as adsorbent, C_0 = 500 ppm. Kinetic curves of (b) pseudo-first-order, (c) pseudo-second-order, and (d) intraparticle diffusion models. ______ *15*

Figure S9: (a) Time-dependent Rhodamine B absorption spectra of PhL, C_0 = 500 ppm. Kinetic curves of (b) pseudo-first-order, (c) pseudo-second-order, and (d) intraparticle diffusion model. ______ *16*

Figure S10: (a) Time-dependent Rhodamine B absorption spectra of CtL, C_0 = 500 ppm. Kinetic curves of (b) pseudo-first-order, (c) pseudo-second-order, and (d) intraparticle diffusion models. ______ *17*

Figure S11: (a) Time-dependent Rhodamine B absorption spectra of HQL, C_0 = 500 ppm. Kinetic curves of (b) pseudo-first-order, (c) pseudo-second-order, and (d) intraparticle diffusion models. ______ *18*

Figure S12: (a) Langmuir and (b) Freundlich adsorption isotherms of Rhodamine B adsorption onto PhL. ______ *18*

Figure S13: (a) Langmuir and (b) Freundlich adsorption isotherms of Rhodamine B adsorption onto CtL. ______ *19*

Figure S14: (a) Langmuir and (b) Freundlich adsorption isotherms of Rhodamine B adsorption onto HQL. ______ *19*

Figure S15. (a) Langmuir and (b) Freundlich adsorption isotherms of Rhodamine B adsorption onto PgL. ______ *19*

Figure S16. Photos used for TOC; from left to right: spruce sawdust, isolated lignin, lignin plus 500 ppm RhB water solution before and after adsorption. ______ *20*

WILEY-VCH

The sorption capacity ____ 20

Figure S17. Sorption capacity expressed as specific surface are (determined by BET) and concentration of the total Ar-OH groups (sum of modifier OH and guaiacyl OH groups measured through 31P NMR) vs equilibrium adsorption capacity. _____________________ 20

WILEY-VCH

Materials

Spruce sawdust (*Picea abies*) of 40-80 years old trees was harvested in Svealand Sweden in 2023, the timber was debarked and processed at AB Karl Hedin Sågverk in Karbenning Sweden. Fresh side streams of spruce sawdust were air dried before storage. All commercial chemicals were analytical reagents and were used without further purification. Phenol (liquified 80:20 w/w in water, general purpose grade, Fischer Chemical), catechol (≥99%, laboratory reagent grade, Fischer Chemical), resorcinol (≥98%, FG, Sigma-Aldrich), pyrogallol (98%+, Alfa Aesar), hydroquinone (99%, Acros Organics), Sulphuric acid, ACS, 95-98% (Alfa Aesar), Hydrochloric acid 37% (Reag. Ph. Eur. VWR), acetone, methanol, ethanol, DMSO-d_6, diethyl ether, endo-*N*-hydroxy-5-norbornene-2,3-dicarboximide (97%, Alfa Aesar), 2-Chloro-4,4,5,5-tetramethyl-1,3,2-dioxaphospholane (95%, Merck), chromium(III) acetylacetonate (97%, Acros organics), Pyridine (anhydrous, 99,8%, Sigma-Aldrich), Chloroform-d, tetrahydrofuran (HPLC grade, unstabilized 99.8%, Fisher). Rhodamine B (Sigma Life Sciences, Dye content 95%), Molecular sieves (type 3A, Merck).

General protocol for synthesis of the organosolv lignin derivatives from biomass (PhL, CtL, ReL, PgL, and HQL)

Synthesis of the organosolv lignin derivatives were carried out following the described procedure with minor modifications. Briefly, in a three-neck round-bottom flask (3 L) equipped with mechanical PTFE propeller stirrer, required amount of dried biomass (300-400 g) and the modifier was pre-soaked overnight in water. The ratio of biomass to solvent was 1:5 w/w (stated solvent ratio includes modifier and water mixture). Modifier is referred as an electron rich aromatic structure bearing at least one hydroxyl group that was introduced to the lignin structure during its extraction from the lignocellulose biomass. In this work, we have used phenol, catechol, resorcinol, hydroquinone, and pyrogallol as modifiers. The reaction conditions for the preparation of organosolv lignin derivatives are presented in table 1. For solid modifiers; e.g., catechol, resorcinol, hydroquinone and pyrogallol, the modifier to water ratio was 1:1 w/w. However, for the phenol modifier, the phenol to water ratio was 3:2 w/w. After presoaking, catalytic amount of 12 M H_2SO_4 (2 wt% of the total solvent amount) was added and the reaction mixture was heated for about 1 h until the temperature reached 100 °C. Then, the reaction mixture was refluxed for 5 h. The reaction mixture was cooled down to ca. 50-60 °C and quenched by addition of methanol (~75 v% of the water amount). The pulp was filtered off and washed with acetone (~75 v% of the water amount) using vacuum filtration. The filtrate was evaporated to remove the acetone and methanol and then poured into a solvent mixture of diethyl ether: water (7:3 v/v). Lignin isolation was not optimized, to obtain maximum possible precipitate diethyl ether amount should be adjusted according to lignin solubility for each modifier. The precipitate thus formed was filtered using vacuum filtration. Thereafter, the dried precipitate was dissolved in acetone and water mixture (7:1 v/v), keeping the total added solvent volume to obtain ca. 0.2 $g\cdot mL^{-1}$ concentration of the precipitate. The resulting solution was added dropwise to vigorously stirred deionized water (5 times the initial solvent volume) to precipitate the organosolv lignin derivatives. Precipitated lignin was collected by vacuum filtration followed by freeze-drying and the samples were named as **PhL**, **CtL**, **ReL**, **PgL**, and **HQL** based on the modifiers used during their extraction, e.g., phenol, catechol, resorcinol, pyrogallol, and hydroquinone respectively. The amount of modified lignin was calculated considering the presence of (26.9+0.4) % lignin in the wood composition based on the previous analysis (Table S1). Therefore, the amount (w%) of obtained modified lignin derivatives based on the starting biomass were ~ 69%, 37%, 46%, 21%, and 27% for **PhL**, **CtL**, **ReL**, **PgL**, and **HQL** respectively.

WILEY-VCH

Table S1. Summary of the reaction conditions for synthesis of the organosolv lignin derivatives from biomass

Sample code	Biomass (spruce sawdust) to solvent ratio wt:wt	Solvent composition (modifier to water, wt:wt)	Catalyst, molarity in solvent	Reaction Condition
**PhL**	1:5	3:2 phenol to water	0.2 M H_2SO_4	Pre-heating 1 h; reflux 5 h
**CtL**	1:5	1:1 catechol to water	0.2 M H_2SO_4	Pre-heating 1 h; reflux 5 h
**ReL**	1:5	1:1 resorcinol to water	0.2 M H_2SO_4	Pre-heating 1 h; reflux 5 h
**PgL**	1:5	1:1 pyrogallol to water	0.2 M H_2SO_4	Pre-heating 1 h; reflux 5 h
**HQL**	1:5	1:1 hydroquinone to water	0.2 M H_2SO_4	Pre-heating 1 h; reflux 5 h

WILEY-VCH

Table S2: Wood composition analysis

Analysis	Unit	Method[a]	Spruce
Arabinose	g/kg DS	SCAN-CM 71:09	10.2
Galactose	g/kg DS	SCAN-CM 71:09	18.0
Glucose	g/kg DS	SCAN-CM 71:09	428
Xylose	g/kg DS	SCAN-CM 71:09	50.3
Mannose	g/kg DS	SCAN-CM 71:09	111
Cellulose	% DS		63.4
Hemicellulose	% DS		36.6
Lignin, Klason	% DS	Tappi T222	26.5
Lignin, Acid-soluble	% DS	Tappi T-UM 250	0.4
Dry substance paper, pulp & board	%	ISO 638	88.7

Wood composition analysis was performed by MoRe Research Örnsköldsvik AB, Örnsköldsvik Sweden. The carbohydrates were analyzed according to the method SCAN-CM 71:09 as described by the Scandinavian Pulp, Paper and Board testing committee in 2009, and were calculated as anhydro sugars. Hemicellulose and cellulose were calculated as % of carbohydrates. Further, the standard methods Tappi T222, Tappi T-UM 250 and ISO 638 were used.

Determination of hydroxyl groups in lignin by quantitative ^{31}P NMR

The hydroxyl content of the organosolv lignin derivatives was determined following the procedure described by X. Meng et al. Briefly, approximately 30 mg of the organosolv lignin derivatives were

WILEY-VCH

weighed directly in 5 mm Wilmad® NMR tubes followed by addition of 0.5 mL anhydrous $CDCl_3$-pyridine (2:3 v/v) solution containing relaxation agent (chromium(III) acetylacetonate, 10 mg·mL^{-1}) and internal standard (endo-N-hydroxy-5-norbornene-2,3-dicarboximide, NHND, 3 mg·mL^{-1}). The samples were then sonicated at RT to ensure complete dissolution. Next, 0.1 mL of the phosphitylating reagent, 2-Chloro-4,4,5,5-tetramethyl-1,3,2-dioxaphospholane (TMDP), was added to the solutions. Samples were analyzed using a JEOL Resonance 400 MHz spectrometer within 24 h from the addition of the phosphitylating reagent. Chemical shifts are reported in ppm relative to the TMDP + H_2O signal (δ = 132.2 ppm). ^{31}P spectra were recorded using 128 scans and relaxation delay of 10 s. Signals were processed in MestreNova software using automatic baseline and phase correction. The content of hydroxyl groups was determined by integrating and comparing with the internal standard, NHND (151.5-152.1 ppm). In the case for the modifiers with more than one hydroxyl groups, the integral area was divided by the number of hydroxyl groups. Details of the different regions for the hydroxyl groups are shown in Table S2, and the quantitative amounts of those hydroxyl groups in different organosolv lignin derivatives are shown in Table S3.

Table S3: Different regions for the hydroxyl groups obtained from ^{31}P NMR spectra of the organosolv lignin derivatives

Organosolv lignin derivatives	Aliphatic hydroxyls (δP)	Guaiacyl hydroxyls (δP)	Modifier hydroxyls (δP)
**PhL**	149.0-146.0	140.0-139.0	138.5-137.0
**PgL**	148.5-145.0	140.0-139.0	144.5-141.5<br>138.5-137.0
**ReL**	149.0-145.2	140.0-139.1	139.1-135.9
**CtL**	148.0-146.5	140.0-139.0	138.9-138.2
**HQL**	149.0-145.4	140.4-139.1	139.1-136.5

WILEY-VCH

Table S4: Quantitative amounts of hydroxyl groups in organosolv lignin derivatives determined from different regions of the ^{31}P NMR spectra

Organosolv lignin derivatives	Aliphatic hydroxyls (mmol/g)	Guaiacyl hydroxyls (mmol/g)	Modifier hydroxyls (mmol/g)
**PhL**	3.15	0.402	3.24
**CtL**	2.19	0.359	3.33
**ReL**	0.434	0.174	1.42
**PgL**	0.179	0.323	2.22
**HQL**	1.191	0.54	1.7

Thermogravimetric Analysis (TGA)

TGA was performed with a TGA/DSC 3+ (Mettler Toledo, Schwerzenbach, Switzerland). Samples with an approximate weight between 5 and 10 mg were heated from 25 to 800 °C under nitrogen flow in uncovered alumina crucibles at a heating rate of 10 K·min^{-1}.

Size Exclusion Chromatography

Approximately 2.5 mg of organosolv lignin derivatives were loaded in Agilent Technologies 0.45 µM PP mini-prep vials. To that, THF (0.5 mL, unstabilized) was added and the samples were sonicated for 10 min to ensure complete solubilization before the filters were pushed down. The samples were then analyzed using a Hitachi HPLC Chromaster system equipped with two LC Phenogel columns 50 Å and 500 Å (5 µm, 7.8×300 mm each) connected in series (flow rate: 1 mL·min^{-1}; injection volume: 10 µL; solvent: THF), with a UV detector (280 nm) and auto-sampler. The system was calibrated using ReadyCal-Kit poly(styrene) (266, 682, 1250, 2280, 3470, 4920, 9130, 15700, 21500, 28000, 44200, 66000 g·mol^{-1}). Determination of M_n and M_w was done using Clarity Chromatography Software.

WILEY-VCH

Table S5. Number average molecular weight (M_n), weight average molecular weight (M_w), and dispersitie**s**, *Đ* (M_n/M_w) of organosolv lignin derivatives.

**Lignin**	**M_n (g·mol^{-1})**	**M_w (g·mol^{-1})**	**Đ**
**PhL**	2500	4200	1.68
**CtL**	3000	4100	1.37
**ReL**	1800	2400	1.33
**PgL**	1900	2800	1.47
**HQL**	2100	3400	1.62

Dynamic Light Scattering

Organosolv lignin derivatives were dispersed in deionized water at a final concentration of 0.5 mg·mL^{-1} for particle size measurements using dynamic light scattering (DLS). The sample preparation procedure was as follows: 25.0 mg of dried organosolv lignin derivatives was transferred into a 50 mL polypropylene centrifuge tube followed by 50.0 mL of deionized water to achieve a final concentration of 0.5 mg·mL^{-1}. The dispersion was sonicated in an ultrasonic bath (40 kHz, 100 W) for 30 min at RT. A portion of the prepared dispersion was transferred into a cuvette. DLS measurements were performed at 25 °C using a Zetasizer 3600 (Malvern). Results are shown in Figures S1–S5.

FT-IR

FT-IR spectra were recorded in ATR mode on a Bruker Tensor 27 ATR-FTIR Spectrometer (Bruker).

Gas sorption

Porosity of the samples was investigated by nitrogen (N_2) sorption at -196 °C on an ASAP 2020 instrument (Micrometrics). The samples were degassed at 150 °C for 10 h under dynamic vacuum prior to the measurement, and the data were analyzed using the Micromeritics MicroActive software. Surface area, SA_{BET}, was calculated using the Brunauer-Emmett-Teller (BET) method, and the density functional theory (DFT) pore size distributions were obtained from the N_2 adsorption isotherms. The total pore volume accessible by N_2, V_{total}, was determined from the adsorption branch of the isotherms at p/p_0 = 0.98.

WILEY-VCH

Table S6. BET surface areas and total pore volumes obtained from N_2 isotherms recorded at -96 °C for the organosolv lignin derivatives.

Lignin	SA_{BET} ($m^2 \cdot g^{-1}$)	V_{tot} ($cm^3 \cdot g^{-1}$)
**PhL**	9.0	0.015
**CtL**	11	0.024
**ReL**	52	0.200
**PgL**	21	0.072
**HQL**	27	0.057

Kinetics and adsorption studies

The efficiency of the as synthesized organosolv lignin derivatives towards removal of cationic dye (Rhodamine B, RhB) at neutral pH was investigated by spectroscopic methods. For the kinetic studies, 500 mg of each material was added to 50 mL of Rhodamine B solution (500 ppm, based on the concentration of the cationic part of the Rhodamine B dye, adjusted for the purity) at neutral pH (7.0–7.2). The solution was shaken in an Orbital Shaker INC/REFRIG 5000IR at 25 °C and 130 rpm. Aliquots were collected at predetermined time intervals. The liquid and solid phases were separated by centrifuging at 3800 × g for 10 min. The residual dye concentration was then measured with an Agilent Cary 60 UV-Vis spectrophotometer at 554 nm. The dye uptake at time *t* (q_t, mg/g), was obtained by converting absorbance to concentration using the calibration curve (Figure S7) and the following formula:

$$q_t = \frac{C_i - C_t}{\mathrm{m}} V \qquad \text{Eq. S1}$$

where, C_i (ppm) is initial dye concentration and C_t (ppm) is the concentration of the dye at time *t*; *m* (g) is the mass of the organosolv lignin derivative and *V* (L) is the volume of the dye solution.
For the isotherm studies, 100 mg of each organosolv lignin derivative was added to dye solutions ranging from 50 to 1500 ppm (based on the cationic part of Rhodamine B, adjusted for purity). After 18 h, concentration of the remaining dye in the solution was measured. The dye uptake per unit weight of lignin (q_e, mg/g) was then calculated using the following formula:

$$q_e = \frac{C_i - C_e}{\mathrm{m}} V \qquad \text{Eq. S2}$$

where, C_i (ppm) is initial dye concentration and C_e (ppm) is the equilibrium concentration; *m* (g) is the mass of the organosolv lignin derivative and *V* (L) is the volume of the dye solution.

WILEY ◆ VCH

Regeneration of the adsorbent

Regeneration of the adsorbent was performed using distilled water as the eluting agent. Dye-loaded adsorbent was immersed in distilled water and sonicated at ~50 °C, then washed and centrifuged until the supernatant became colorless. The recovered adsorbent was oven-dried before reuse. Adsorption–desorption cycles were conducted with 200 ppm dye solution, and the percentage of dye removed (*R*) after each cycle was calculated using the following formula:

$$R = \frac{C_i - C_e}{C_i}\, 100\% \qquad \text{Eq. S3}$$

where, C_i (ppm) is initial dye concentration and C_e (ppm) is the equilibrium concentration.

SEM

Morphology of the dried samples before and after dye adsorption was studied using a Zeiss LEO 1530 scanning electron microscope (SEM) (Oberkochen, Germany) equipped with a Schottky FEG and operated at 2.5 kV. The samples were Au sputter-coated prior to imaging. The images were analyzed using ImageJ software.

Table S7: Values of $T_{d50\%}$ (°C), Residue (%) and T_{max} (°C) for the lignin derivatives

Sample	$T_{d50\%}$ (°C)[a]	Residue (%)[a]	T_{max} (DTG)[b] (°C)
PhL	410	32	383
CtL	421	36	358
ReL	530	41	350
PgL	445	37	327
HQL	410	33	367

[a] Obtained from TGA and [b] obtained from DTG analysis

WILEY-VCH

			Size (d.nm):	% Intensity:	St Dev (d.nm):
Z-Average (d.nm):	1267	Peak 1:	562.1	42.9	107.7
PdI:	0.902	Peak 2:	933.7	41.6	184.8
Intercept:	0.911	Peak 3:	253.2	15.5	47.59

Result quality : Refer to quality report

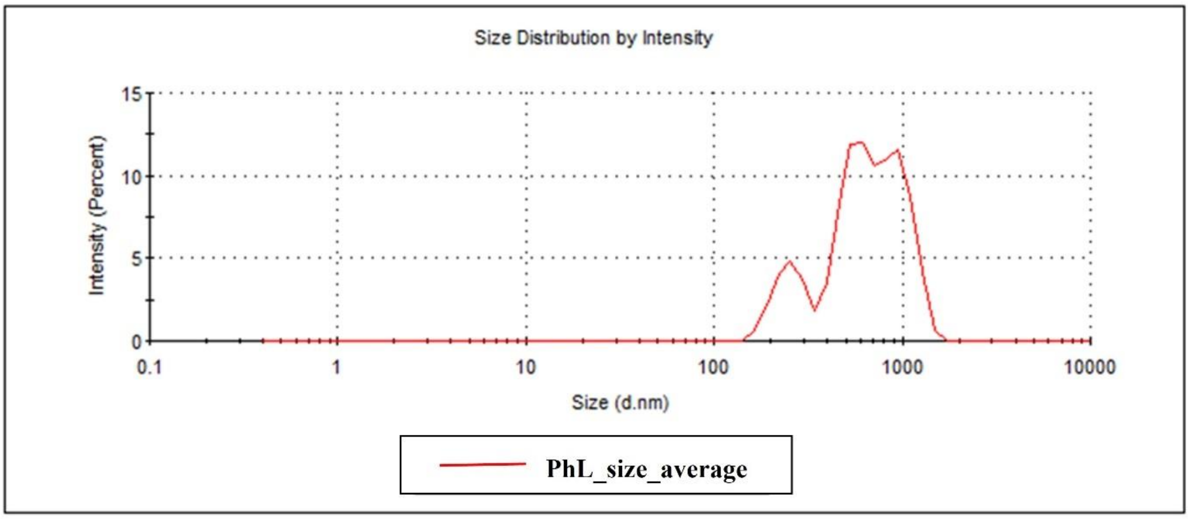

Figure S1: Intensity-weighted particle size distribution of PhL measured by DLS at room temperature.

			Size (d.nm):	% Intensity:	St Dev (d.nm):
Z-Average (d.nm):	872.2	Peak 1:	592.5	90.9	300.2
PdI:	0.698	Peak 2:	5408	9.1	303.4
Intercept:	0.865	Peak 3:	0.000	0.0	0.000

Result quality : Refer to quality report

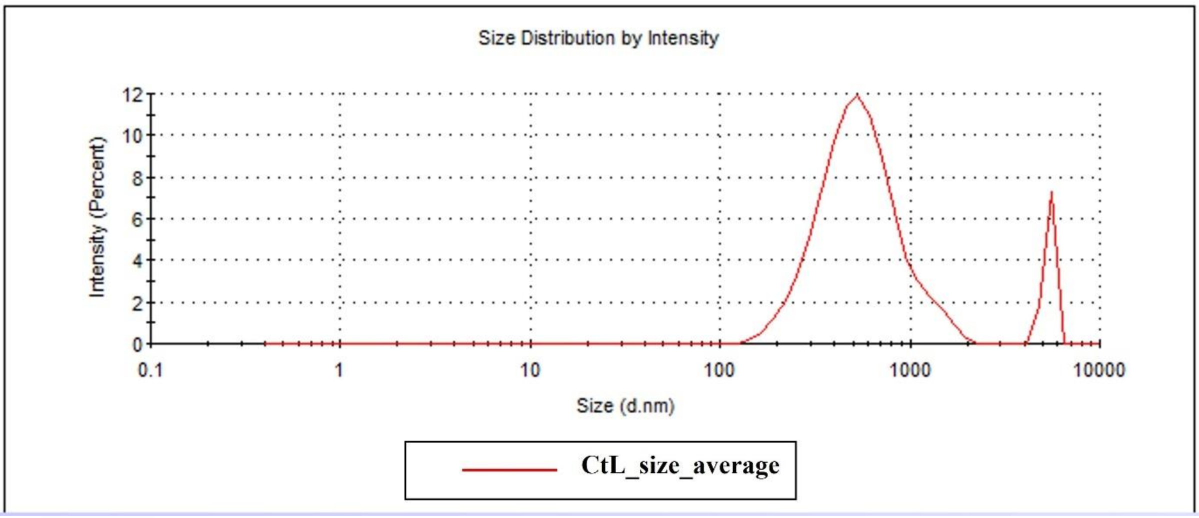

Figure S2: Intensity-weighted particle size distribution of CtL measured by DLS at room temperature.

WILEY-VCH

			Size (d.nm):	% Intensity:	St Dev (d.nm):
Z-Average (d.nm):	285.9	Peak 1:	341.6	93.6	196.3
PdI:	0.332	Peak 2:	4321	6.4	1072
Intercept:	0.916	Peak 3:	0.000	0.0	0.000
Result quality :	Good				

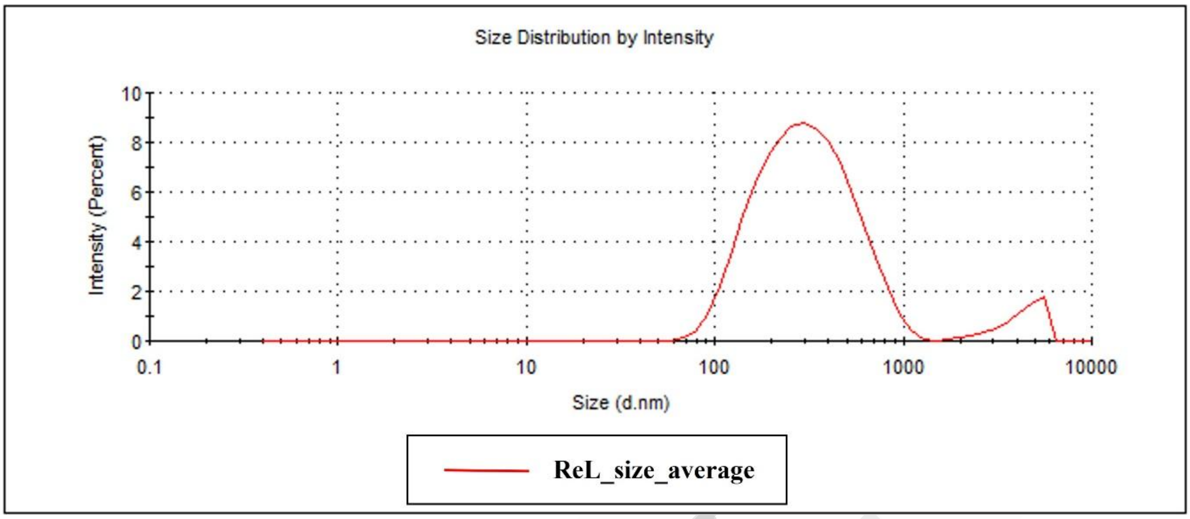

Figure S3: Intensity-weighted particle size distribution of **ReL** measured by DLS at room temperature.

			Size (d.nm):	% Intensity:	St Dev (d.nm):
Z-Average (d.nm):	352.7	Peak 1:	404.6	94.6	195.0
PdI:	0.273	Peak 2:	4317	5.4	1035
Intercept:	0.814	Peak 3:	0.000	0.0	0.000
Result quality :	Good				

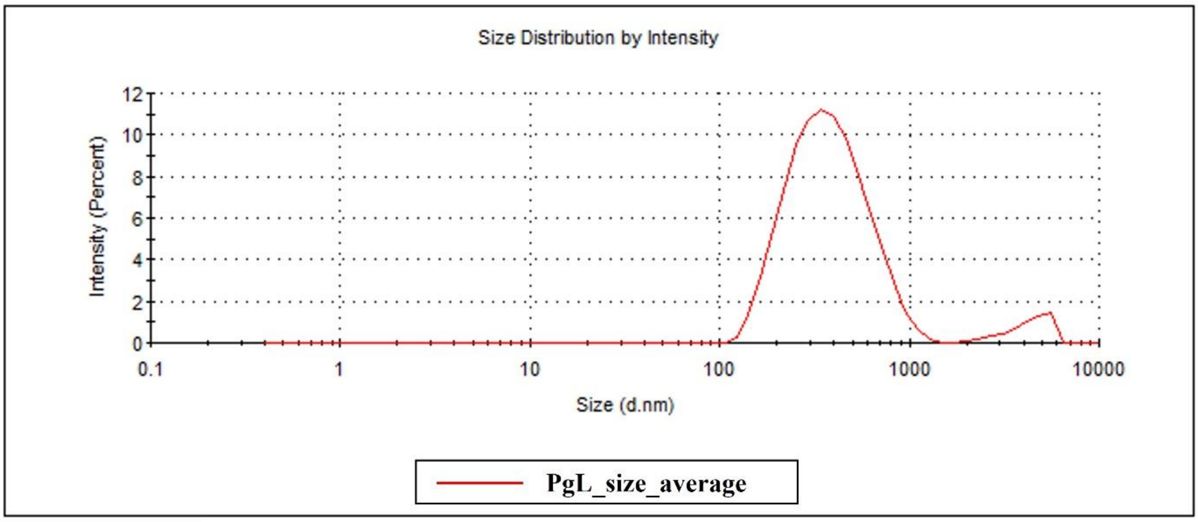

Figure S4: Intensity-weighted particle size distribution of **PgL** measured by DLS at room temperature.

			Size (d.nm):	% Intensity:	St Dev (d.nm):
Z-Average (d.nm):	497.0	Peak 1:	964.2	100.0	961.9
PdI:	0.405	Peak 2:	0.000	0.0	0.000
Intercept:	0.551	Peak 3:	0.000	0.0	0.000
Result quality :	Good				

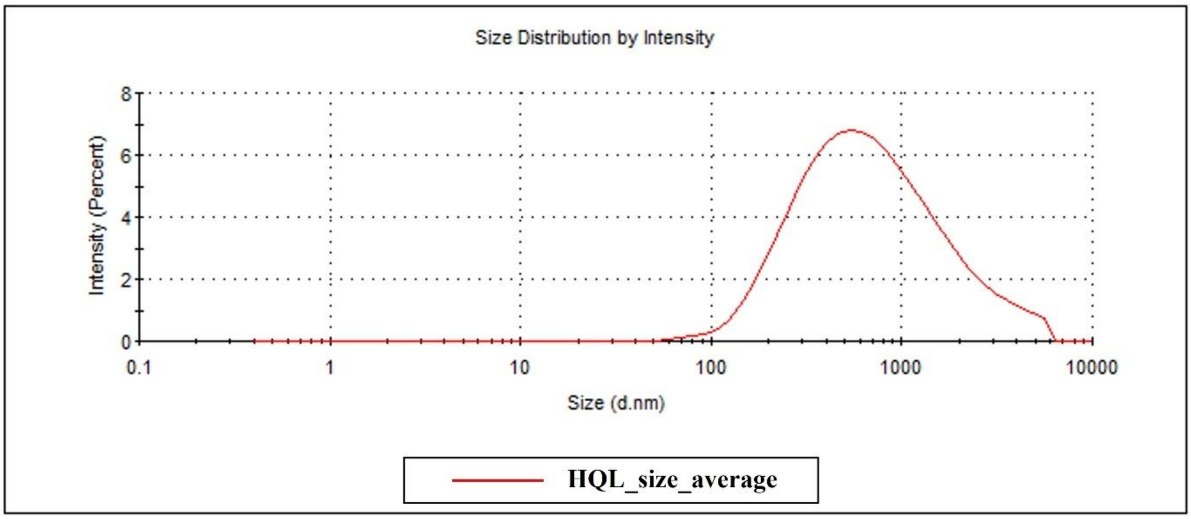

Figure S5: Intensity-weighted particle size distribution of HQL measured by DLS at room temperature.

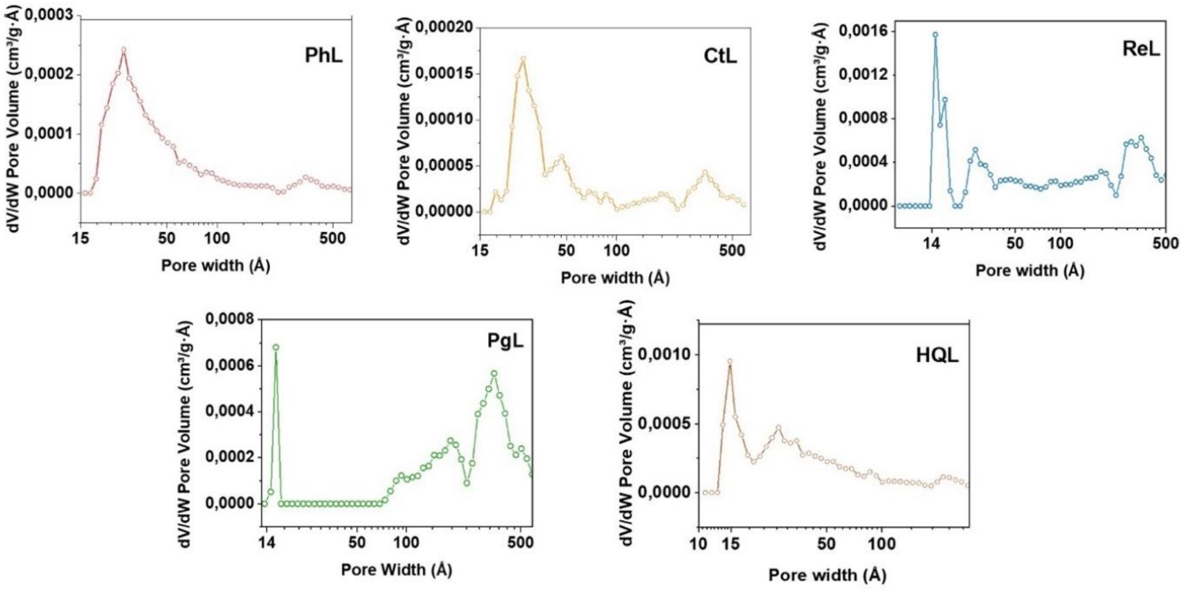

Figure S6: Pore-size distributions of **PhL**, **CtL**, **ReL**, **PgL**, and **HQL** obtained from BET analysis.

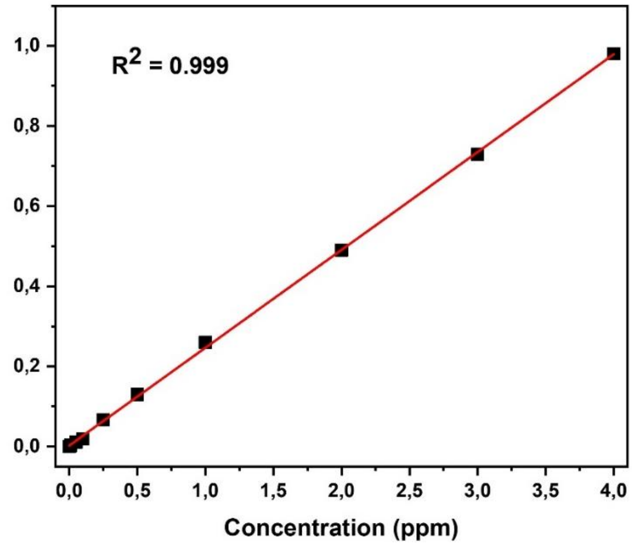

Figure S7: Calibration curve of the Rhodamine B dye.

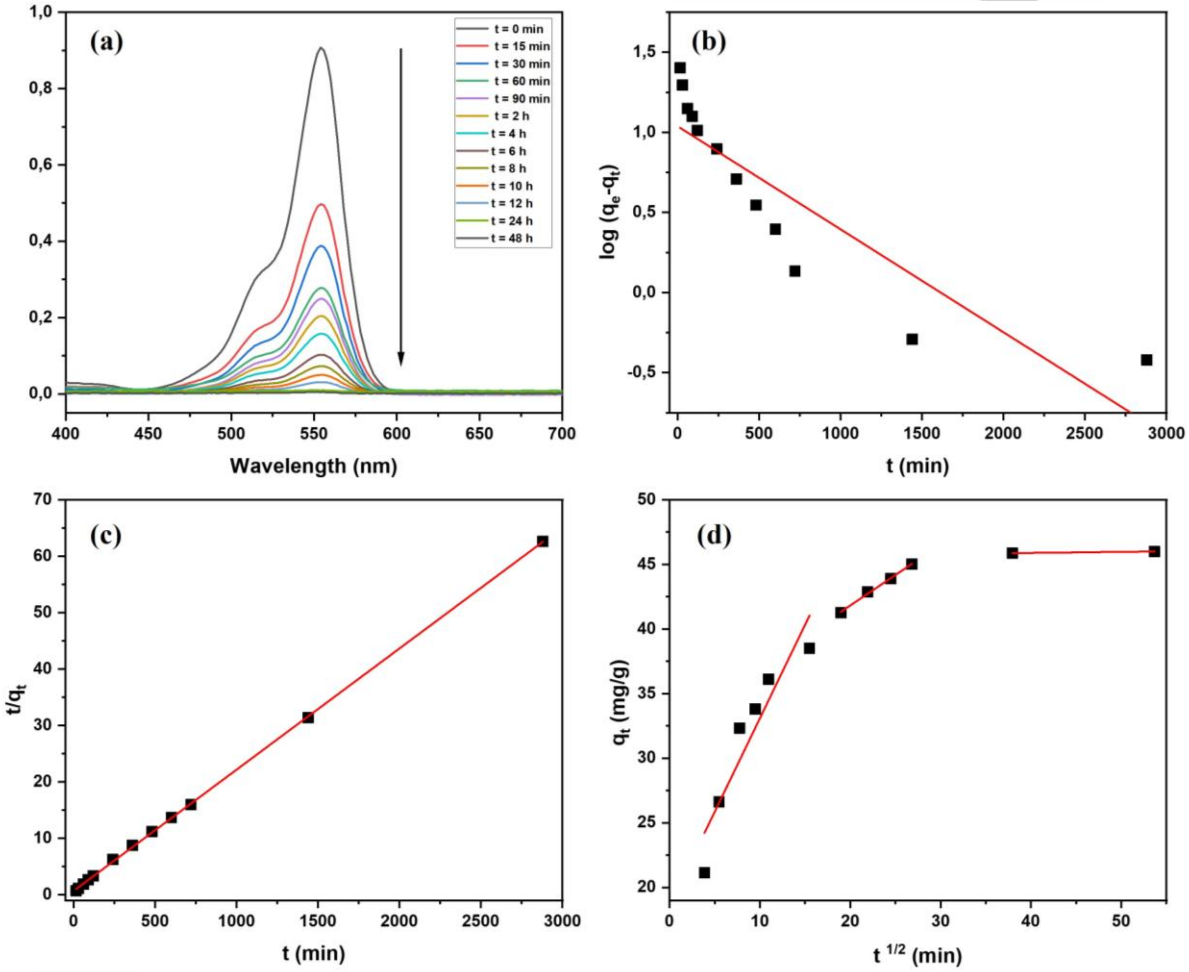

Figure S8. (a) Time-dependent Rhodamine B absorption spectra using **PgL** as adsorbent, C_0 = 500 ppm. Kinetic curves of (b) pseudo-first-order, (c) pseudo-second-order, and (d) intraparticle diffusion models.

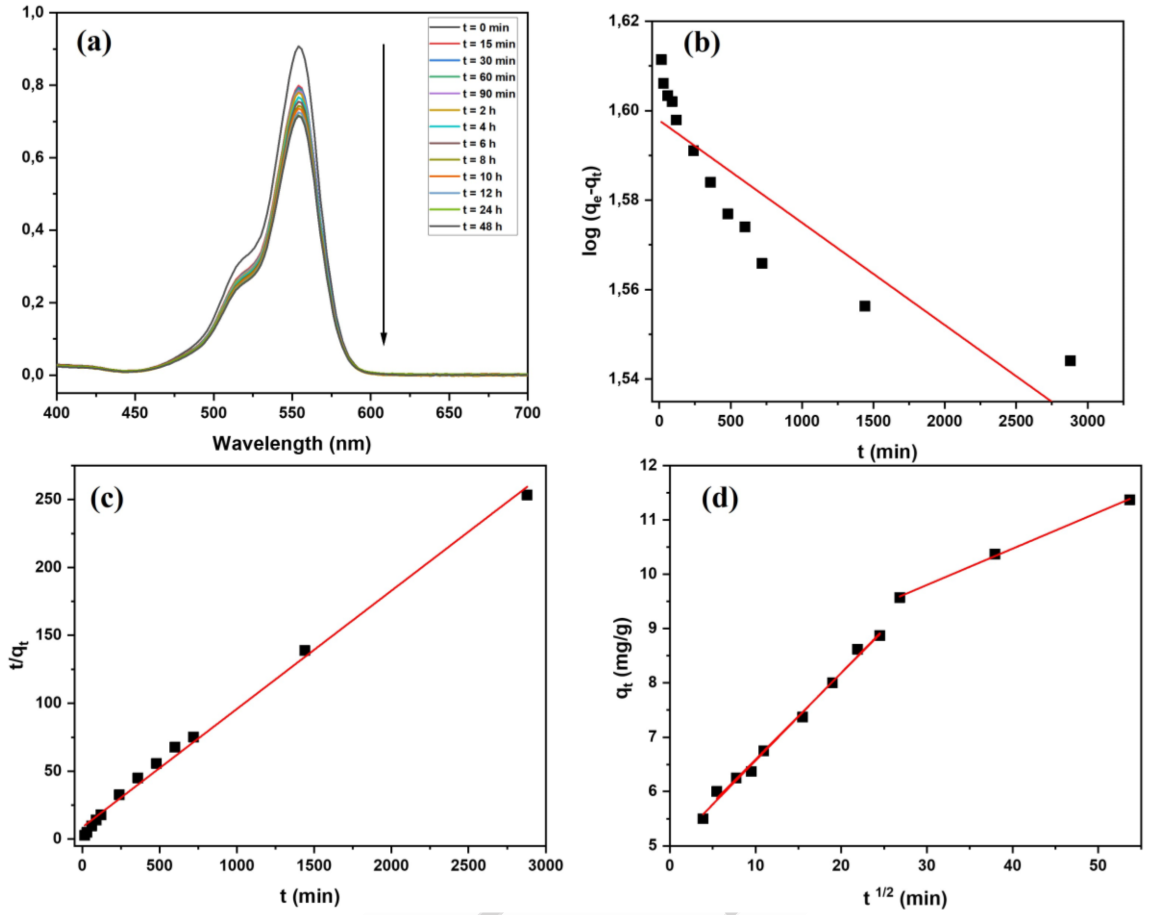

Figure S9: (a) Time-dependent Rhodamine B absorption spectra of PhL, C_0 = 500 ppm. Kinetic curves of (b) pseudo-first-order, (c) pseudo-second-order, and (d) intraparticle diffusion model.

WILEY

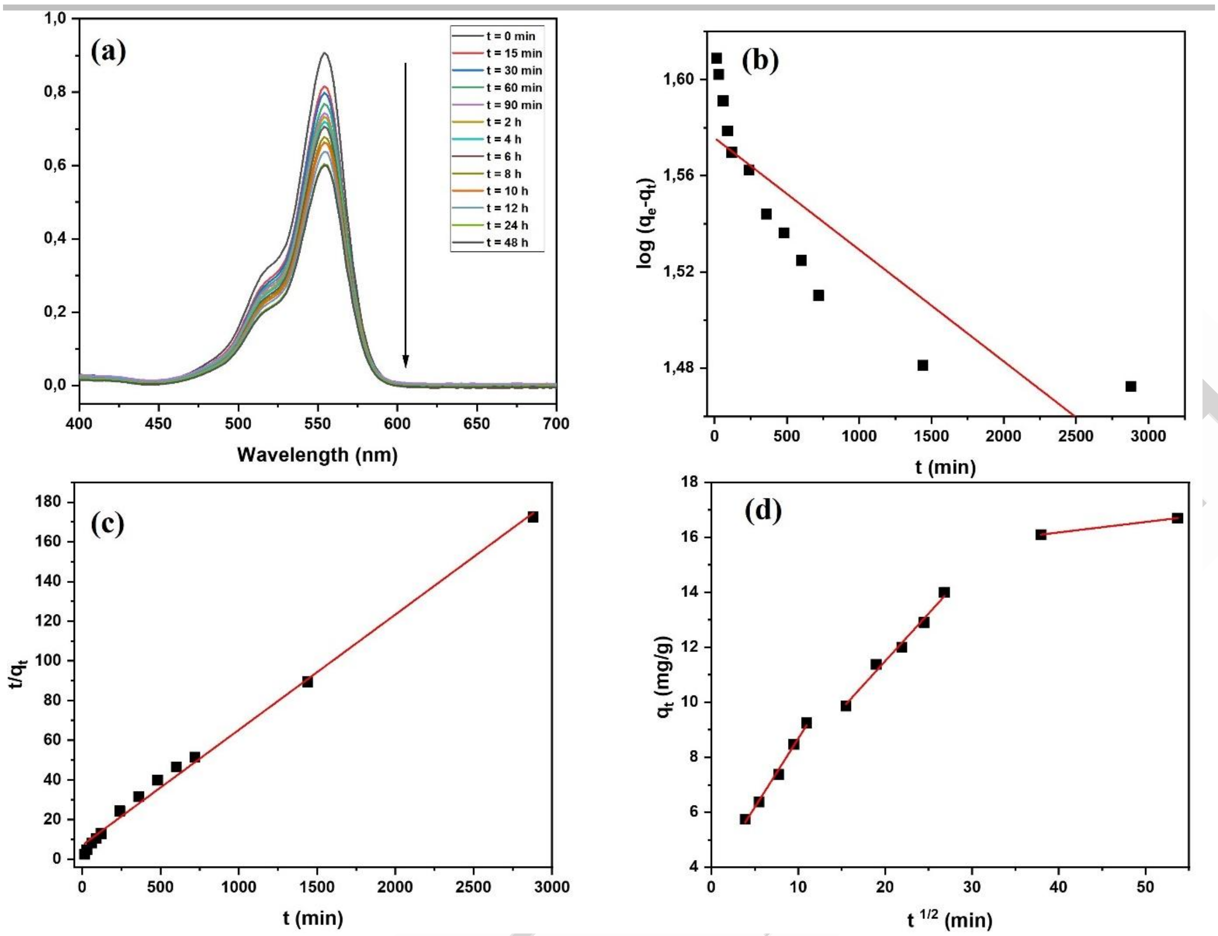

Figure S10: (a) Time-dependent Rhodamine B absorption spectra of **CtL**, C_0 = 500 ppm. Kinetic curves of (b) pseudo-first-order, (c) pseudo-second-order, and (d) intraparticle diffusion models.

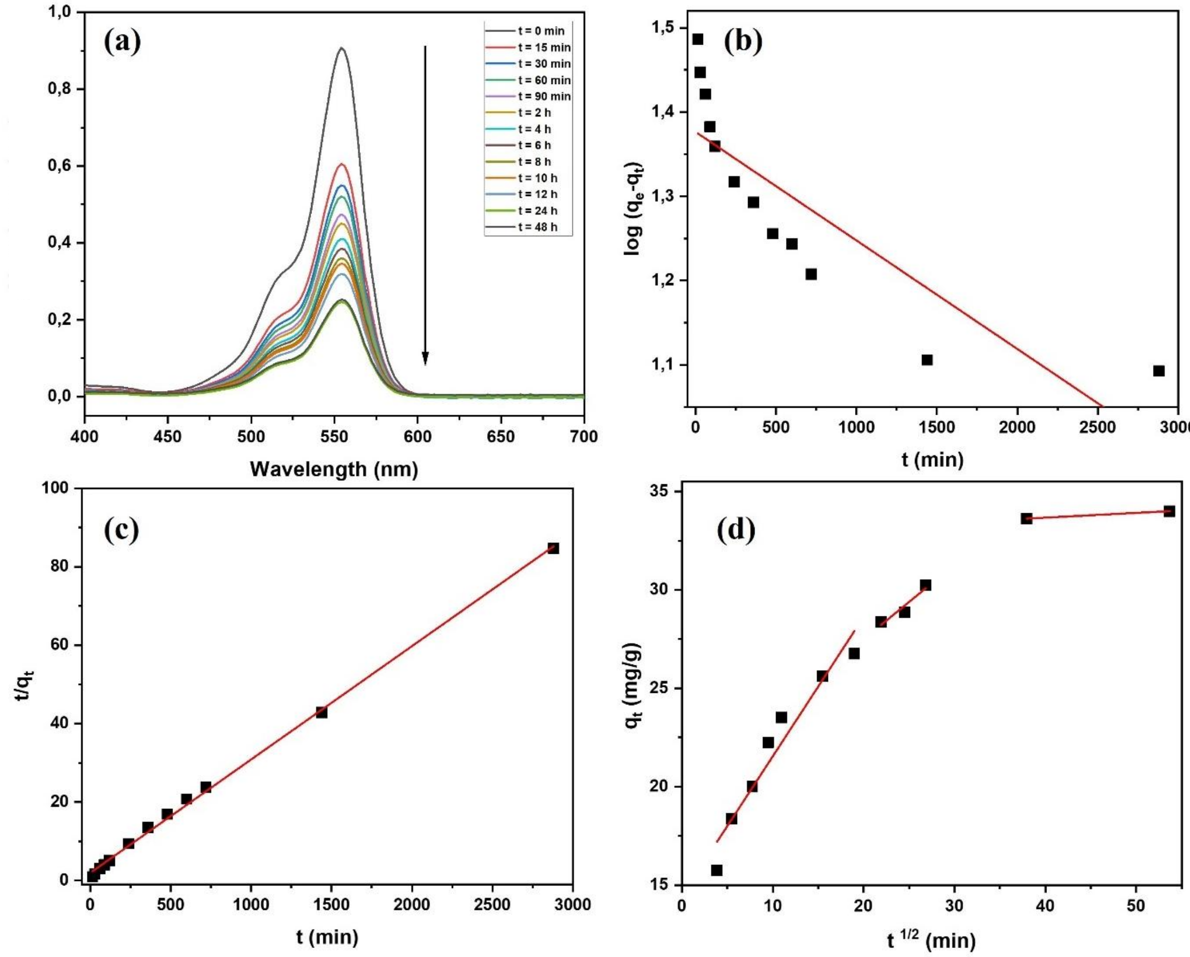

Figure S11: (a) Time-dependent Rhodamine B absorption spectra of **HQL**, C_0 = 500 ppm. Kinetic curves of (b) pseudo-first-order, (c) pseudo-second-order, and (d) intraparticle diffusion models.

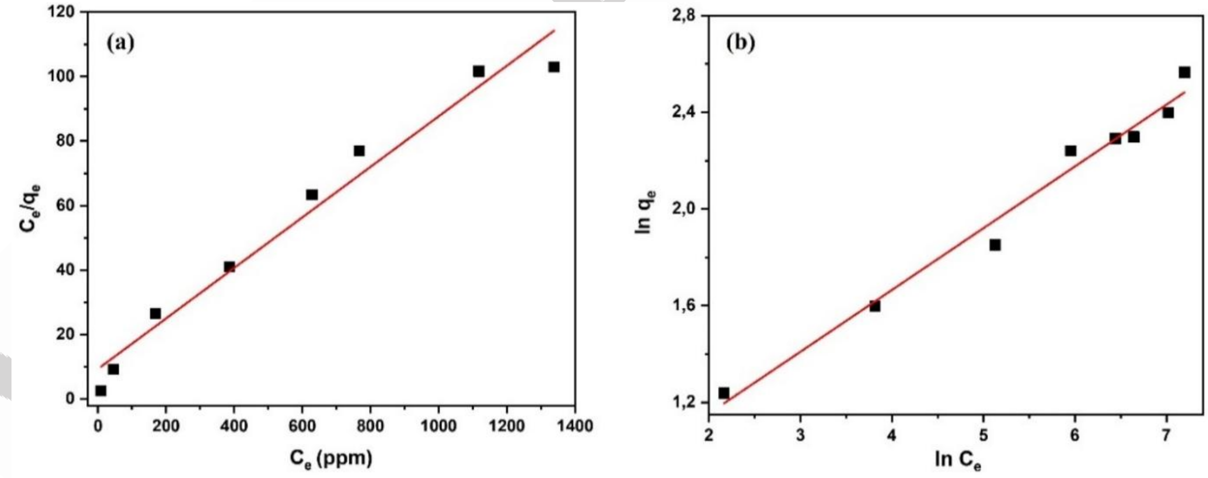

Figure S12: (a) Langmuir and (b) Freundlich adsorption isotherms of Rhodamine B adsorption onto **PhL**.

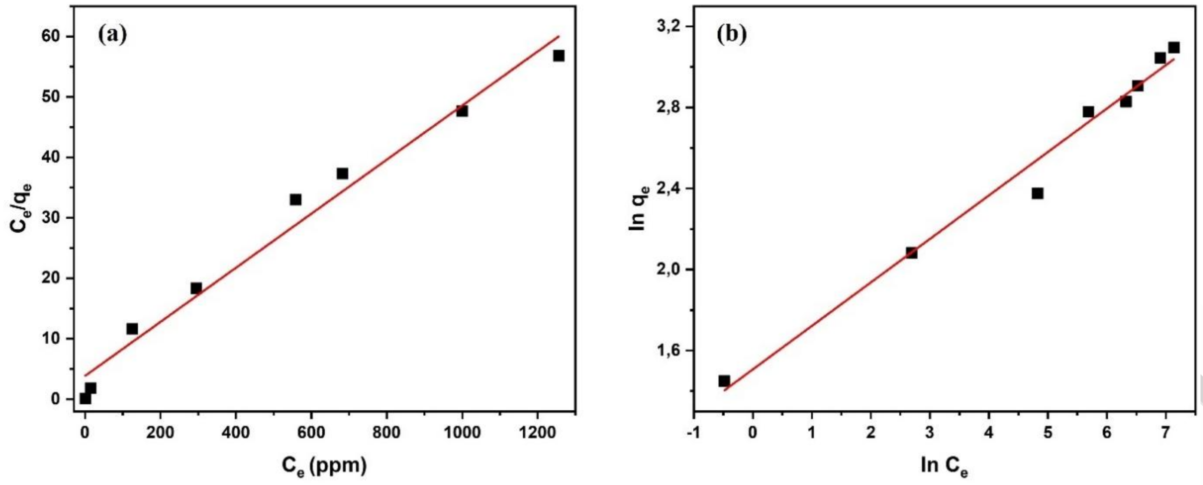

Figure S13: (a) Langmuir and (b) Freundlich adsorption isotherms of Rhodamine B adsorption onto **CtL**.

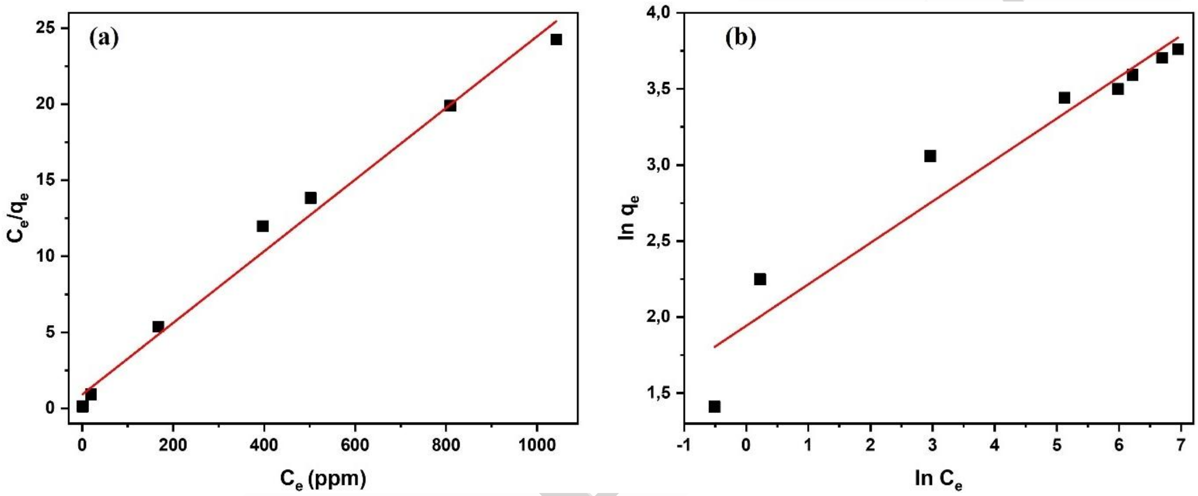

Figure S14: (a) Langmuir and (b) Freundlich adsorption isotherms of Rhodamine B adsorption onto **HQL**.

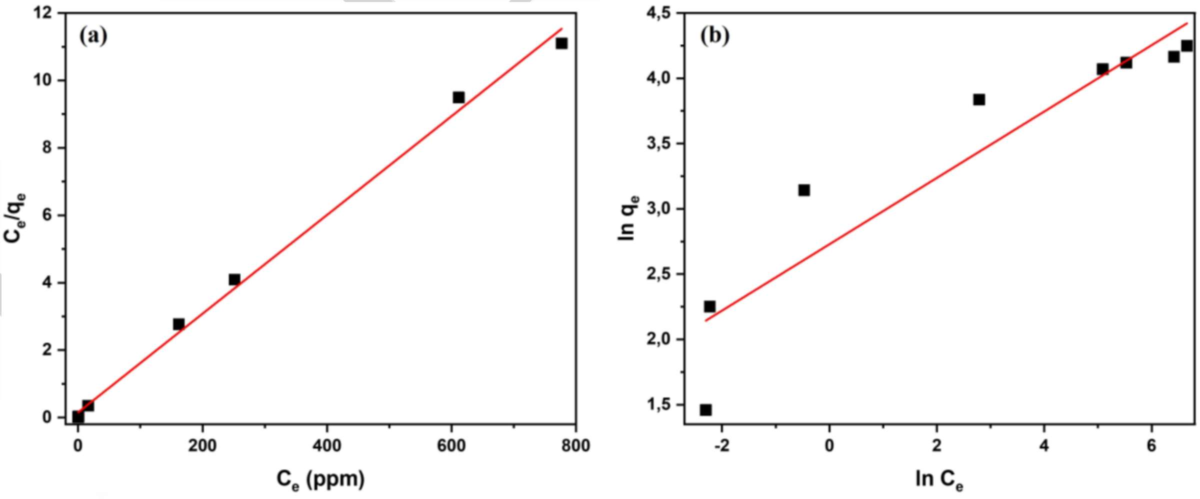

Figure S15. (a) Langmuir and (b) Freundlich adsorption isotherms of Rhodamine B adsorption onto **PgL**.

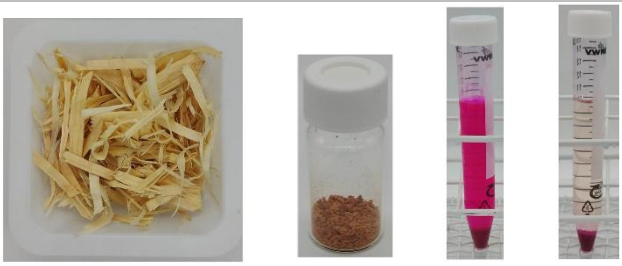

Figure S16. Photos used for TOC; from left to right: spruce sawdust, isolated lignin, lignin plus 500 ppm RhB water solution before and after adsorption.

The sorption capacity

The sorption capacity, i.e., the amount of pollutant a material can uptake, is governed by several physicochemical factors. It is proportional to the specific surface area ($q \propto S_{BET}$), as a larger accessible surface provides more adsorption sites, and to the density of active sites ($q \propto N_{sites}$) in the case of chemisorption, where the concentration of reactive functional groups (e.g., aromatic –OH) determines the uptake potential. Overall, the maximum adsorption capacity can be expressed as $q \propto S_{eff} \times N_{active\ groups}$, where S_{eff} represents the accessible surface area. Consistent with this relationship, our results show that samples possessing both the highest surface area and the largest number of phenolic –OH groups exhibit the highest maximum adsorption capacity, as illustrated in Figure S17.

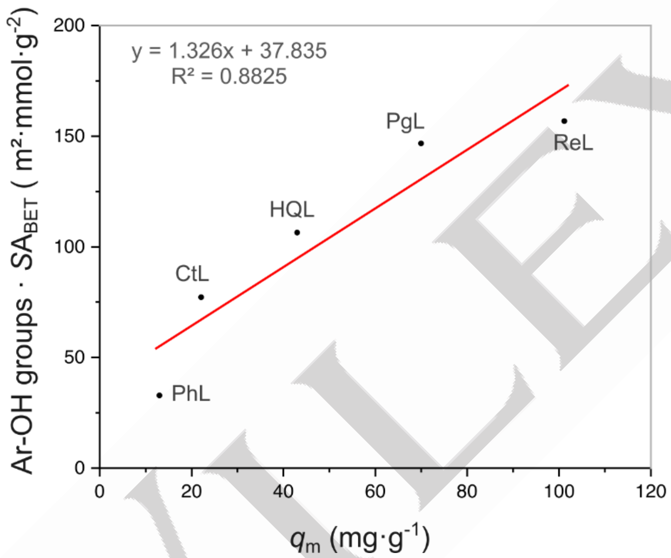

Figure S17. Sorption capacity expressed as specific surface are (determined by BET) and concentration of the total Ar-OH groups (sum of modifier OH and guaiacyl OH groups measured through 31P NMR) vs maximum adsorption capacity.

WILEY • VCH
